# Spatial and temporal clustering analysis of tuberculosis in the mainland of China at the prefecture level, 2005–2015

**DOI:** 10.1186/s40249-018-0490-8

**Published:** 2018-10-20

**Authors:** Meng-Yang Liu, Qi-Huan Li, Ying-Jie Zhang, Yuan Ma, Yue Liu, Wei Feng, Cheng-Bei Hou, Endawoke Amsalu, Xia Li, Wei Wang, Wei-Min Li, Xiu-Hua Guo

**Affiliations:** 1 Department of Epidemiology and Health Statistics, School of Public Health, Capital Medical University, Beijing, 100069 China; 2 Beijing Municipal Key Laboratory of Clinical Epidemiology, Capital Medical University, Beijing, 100069 China; 3 Chinese Center for Disease Control and Prevention, Beijing, 102206 China; 4 Department of Mathematics and Statistics, La Trobe University, Melbourne, 3086 Australia; 5 School of Medical Sciences and Health, Edith Cowan University, WA6027, Perth, Australia; 6 National Tuberculosis Clinical Laboratory of China, Beijing Chest Hospital, Capital Medical University, Beijing, 101149 China; 7 Beijing Tuberculosis and Thoracic Tumour Research Institute, Beijing, 101149 China

**Keywords:** Tuberculosis, Space-time cluster, SaTscan, Prefecture, China

## Abstract

**Background:**

Tuberculosis (TB) is still one of the most serious infectious diseases in the mainland of China. So it was urgent for the formulation of more effective measures to prevent and control it.

**Methods:**

The data of reported TB cases in 340 prefectures from the mainland of China were extracted from the China Information System for Disease Control and Prevention (CISDCP) during January 2005 to December 2015. The Kulldorff’s retrospective space-time scan statistics was used to identify the temporal, spatial and spatio-temporal clusters of reported TB in the mainland of China by using the discrete Poisson probability model. Spatio-temporal clusters of sputum smear-positive (SS+) reported TB and sputum smear-negative (SS-) reported TB were also detected at the prefecture level.

**Results:**

A total of 10 200 528 reported TB cases were collected from 2005 to 2015 in 340 prefectures, including 5 283 983 SS- TB cases and 4 631 734 SS + TB cases with specific sputum smear results, 284 811 cases without sputum smear test. Significantly TB clustering patterns in spatial, temporal and spatio-temporal were observed in this research. Results of the Kulldorff’s scan found twelve significant space-time clusters of reported TB. The most likely spatio-temporal cluster (*RR* = 3.27, *P* <  0.001) was mainly located in Xinjiang Uygur Autonomous Region of western China, covering five prefectures and clustering in the time frame from September 2012 to November 2015. The spatio-temporal clustering results of SS+ TB and SS- TB also showed the most likely clusters distributed in the western China. However, the clustering time of SS+ TB was concentrated before 2010 while SS- TB was mainly concentrated after 2010.

**Conclusions:**

This study identified the time and region of TB, SS+ TB and SS- TB clustered easily in 340 prefectures in the mainland of China, which is helpful in prioritizing resource assignment in high-risk periods and high-risk areas, and to formulate powerful strategy to prevention and control TB.

**Electronic supplementary material:**

The online version of this article (10.1186/s40249-018-0490-8) contains supplementary material, which is available to authorized users.

## Multilingual abstracts

Please see Additional file 1 for translations of the abstract into the six official working languages of the United Nations.

## Background

Tuberculosis (TB) remains one of the most severe infectious disease worldwide with about 10.4 million new cases in 2016 [1]. Especially in China, there were about 0.8 million incident cases reported which alone contributed to 12% of the global reported TB incident with the number of 6.3 million. Additionally, the number of new TB cases in China was just less than India and Indonesia, ranking the third place worldwide in 2016 [1]. Though great achievements have been made for TB control work in recent two decades [2], it was still difficult to achieve the common aim of World Health Organization (WHO)'s End TB - to end the global TB epidemic for the period of 2016–2035. So the precise clustering results in spatial, temporal and spatio-temporal of TB would be helpful to renew national TB strategy of prevention and control.

TB is an airborne infectious disease with spatial autocorrelation in distribution at the international, national, provincial and even smaller levels during certain periods of time. A region with a high risk of TB would affect its neighboring areas. Global and local Moran’s *I* spatial autocorrelation analysis are the commonly used methods to detect whether there exist spatial autocorrelations and where the particular areas are located, separately. However, these methods could only evaluate the distribution characteristic of the disease in specific time point but unable to analyse continuous time [3–5]. As we all know, time is a non-negligible factors that might bias the conclusion directly. The Kulldorff’s space-time scan statistics is a more comprehensive method that could take both spatial and temporal distribution into consideration and set parameters more flexibly. More and more epidemiology studies of infectious disease have used this method widely [6–8]. In China, several studies about the spatio-temporal distribution of TB just in a specific province/municipality such as Beijing [9], Yunnan [10], Qinghai [11] or in the nationwide but at the provincial level [12] were analysed. Therefore, cluster analysis of nationwide TB at a more precise level is urgently needed. And study on every prefecture in the mainland of China has not been reported, so we carry out our research at the prefecture level.

Moreover, previous studies only took all TB cases as a whole and didn’t distinguish sputum smear-positive (SS+) reported TB and sputum smear-negative (SS-) reported TB. SS+ TB not only can seriously damage the patient’s own health, but also can infect others. Studies found that about 70% of individuals with SS+ TB died without regular treatment within 10 years of being diagnosed [1]. Besides, the strong infectivity of SS+ TB can easily cause the outbreak in the crowd gathering area. Though the infectivity of SS- TB is weaker than that of SS+ TB, SS- TB is difficult to diagnose and can revert back to SS+ TB without standard treatment [13], which virtually adds the difficulty to control and eliminate it. Therefore, we need to pay much attention to both SS+ TB and SS- TB.

Analyzing and evaluating the spatio-temporal patterns of TB in the mainland of China at the prefecture level is necessary for TB control and elimination. The aim of our study was to use the Kulldorff’s space-time scan statistical method to explore the spatial, temporal and spatio-temporal distribution characteristics of reported TB at the prefecture level in the mainland of China from 2005 to 2015. What’s more, spatio-temporal analysis of SS+ reported TB and SS- reported TB were also detected to summarize their variation laws.

## Methods

### TB data

Data of TB cases in 340 prefectures in the mainland of China (excluding Taiwan, Hong Kong and Macao) from 2005 to 2015 were collected from the China Information System for Disease Control and Prevention (CISDCP). Three hundred forty prefectures included 287 prefecture-level cities, 30 autonomous prefectures, 17 regions, 3 leagues and 3 provincial-controlled divisions, which covered the entire mainland of China (Additional file 2). Demographic data of every city in every year were extracted from China statistical yearbooks (2006–2016) (http://www.stats.gov.cn/tjsj/ndsj/). TB is one of the most serious infectious diseases in China. It is mandated that each case of TB must be reported online within 24 h after diagnosis. Cases of TB were diagnosed using radiography, pathogen detection, and pathologic diagnosis, based on the diagnostic criteria recommended by the National Health and Family Planning Commission of China.

A total of 10 271 169 incident cases of TB were reported across 2921 hospitals and medical institutions in the mainland of China from January 2005 to December 2015. In this study, 10 200 528 cases aggregated at the prefecture-level city monthly were analyzed to detect the spatio-temporal high-risk areas of TB. And 70 641 cases without detailed information on the residential address were excluded from the analysis. The annual average reported cases were 927 321 during 2005 to 2015. And the percentage of the reported cases included in this study were 99.31%. In addition, the 10 200 528 TB cases included 5 283 983 SS- TB cases and 4 631 734 SS+ TB cases with specific sputum smear results, 284 811 cases without sputum smear test. The missing value percentage of the sputum smear test result was 2.79%. Of course, there were often outbreaks of tuberculosis in some regions with high TB incidence in almost every year especially in schools [14–16].

### Statistical methods

Firstly, Global Moran’s *I* index was used to determine whether there is a global spatial autocorrelation between 340 prefectures. The value of Global Moran’s *I* varies between − 1 and 1. A higher positive Moran’s *I* indicates that values in neighboring positions tend to cluster, while a lower negative Moran’s *I* implies that higher and lower values are interspersed. When Moran’s *I* is near 0, there is no spatial clustering, meaning that the data are randomly distributed [17]. The global spatial autocorrelation analysis was conducted by the ArcGIS 10.2 software (ESRI Inc. Redlands, CA, USA) using the packages of Spatial Autocorrelation. *Z*-score and *P*-value were calculated to evaluate the significance of Global Moran’s *I*.

Then, Kulldorff’s space-time scan statistical analysis was used to explore the temporal, spatial, and spatial-temporal clusters of TB as well as to verify whether the geographic clustering of TB was caused by random variation or not [18]. The discrete poisson probability model was used for scanning since the TB incidence was not very high [19]. We used the radius of the population coverage instead of the geographical radius in this study because the population in several areas was very small [11]. The space-time scan statistics were defined by a cylindrical window with a circular geographic base and with height corresponding to time. Then the cylindrical window was moved in space and time so that for each potential geographical location and size it also visited each possible time period. For each location and size of the scanning window, the alternative hypothesis was that there was an elevated risk within the window as compared to the outside [8]. The most likely cluster is the window with the maximum likelihood while the rest clusters with statistically significant log-likelihood ratios (LLR) were defined as the secondary likely clusters. The *P* value of LLR can be estimated using Monte Carlo method and the number of replications was limited to 999 [20]. The relative risk (*RR*) is the estimated risk within the cluster divided by the estimated risk outside the cluster [21]. In mathematical notation, it is:

$$ RR=\frac{c/E\left[c\right]}{\left(C-c\right)/\Big(E\left[C\right]-E\left[c\right]}=\frac{c/E\left[c\right]}{\left(C-c\right)/\left(C-E\left[c\right]\right)} $$

Where *c* is the number of observed cases within the cluster and C is the total number of cases in the data set. Note that since the analysis is conditioned on the total number of cases observed, *E[C]* = *C*.

The selection of the maximum radius of the spatial scanning window and the maximum length of the temporal scanning window were very important since the results of spatio-temporal scan are sensitive to them [22]. The default setting of the window sizes and temporal sizes were usually set as 50% but some studies questioned whether it is suitable [23]. For example, a high false positive rate might emerge if the window size is too large because some low-risk areas were included. Similarly, a high false negative rate would emerge if the window size is too small [11]. Many researches have explored how to choose an appropriate scanning window, and the mainly rules of those studies were reducing the overlapping areas or a single cluster should no more than 15% of the whole study area when using the irregular scan statistic [24, 25]. And some studies also used this standards in regular scan statistics [11]. So we used this experience for reference in our study. In order to select the optimal parameter of spatial cluster sizes, we analyzed the data of 2005 setting the maximum sizes from 4 to 50% of total population at risk by increments of 1%. If there were fewer overlaps between the areas defined by the radius, or the biggest area covered less than 15% of all the prefectures [11, 24, 25], the radius was considered as an optimal radius for analysis. When the maximum sizes set 4% to 11%, there are fewer overlaps and the biggest area covered no more than 15% of all the prefectures. The number of overlaps ranged from 20 to 26 and the number of prefectures covered by the biggest areas is 23. When the maximum sizes set from 12 to 19%, the number of overlaps reached the biggest of 30. And when the maximum sizes reached and exceeded 20%, the number of prefectures covered by the biggest areas increased more than 15% (51 prefectures) of all the prefectures. So the maximum spatial cluster size was set as 11% eventually. As for the temporal windows, if we set it as the default of 50%, both the pure temporal analysis and spatio-temporal analysis showed the clustering time from the early of 2005 to 2010. The results were not precise and specific, and a high false positive rate might emerge. So we must choose a more optimal temporal window in this study. Previous studies in China reported [10, 11, 26] that the temporal cluster of TB incidents were mainly concentrated in the spring and early summer, so some studies set 30% as the temporal window to do clustering analysis, which means that the maximum scan time length was 3 months for each year. Time series analysis of our study also found the epidemic characteristics of TB with the high incidence were from March to May. Based on the comprehensive summary of previous studies and the temporal characteristics of our study, the maximum temporal cluster size was set as 30% in this study in order to get closer to the real situation in China. Finally, we selected the spatial window covering 11% of the population at risk and the temporal window covering 30% of the whole study period to do our research. The statistical analysis was conducted by the SaTScan 9.3 software (https://www.satscan.org/) and *P*-value less than 0.05 were considered statistically significant.

In addition, time series seasonal decomposition analysis was conducted to identify the seasonality of TB incidence in the mainland of China [27–29]. The time series of reported TB cases were decomposed into seasonal variation, long-term trend and random effect to explore the temporal patterns.

## Results

### Global spatial autocorrelation analysis by global Moran’s *I* index

Significant global spatial autocorrelation existed in the reported TB cases in every year and the average time from 2005 to 2015 (all *P* <  0.001) with the Moran’s *I* index ranging from 0.144 to 0.289. (Table 1). So further spatio-temporal clustering analysis of TB were needed.

**Table 1:** Global spatial autocorrelation analysis of reported tuberculosis in the mainland of China from 2005 to 2015

Year	Moran’s *I* index	*Z*-score	*P*-value
2005	0.144	11.037	< 0.001
2006	0.155	11.797	< 0.001
2007	0.172	13.073	< 0.001
2008	0.179	13.559	< 0.001
2009	0.206	15.516	< 0.001
2010	0.246	18.571	< 0.001
2011	0.289	21.724	< 0.001
2012	0.250	18.841	< 0.001
2013	0.224	16.951	< 0.001
2014	0.215	16.337	< 0.001
2015	0.218	16.652	< 0.001
2005–2015	0.236	17.808	< 0.001

### Distribution of reported TB spatial clustering

**Fig. 1:**
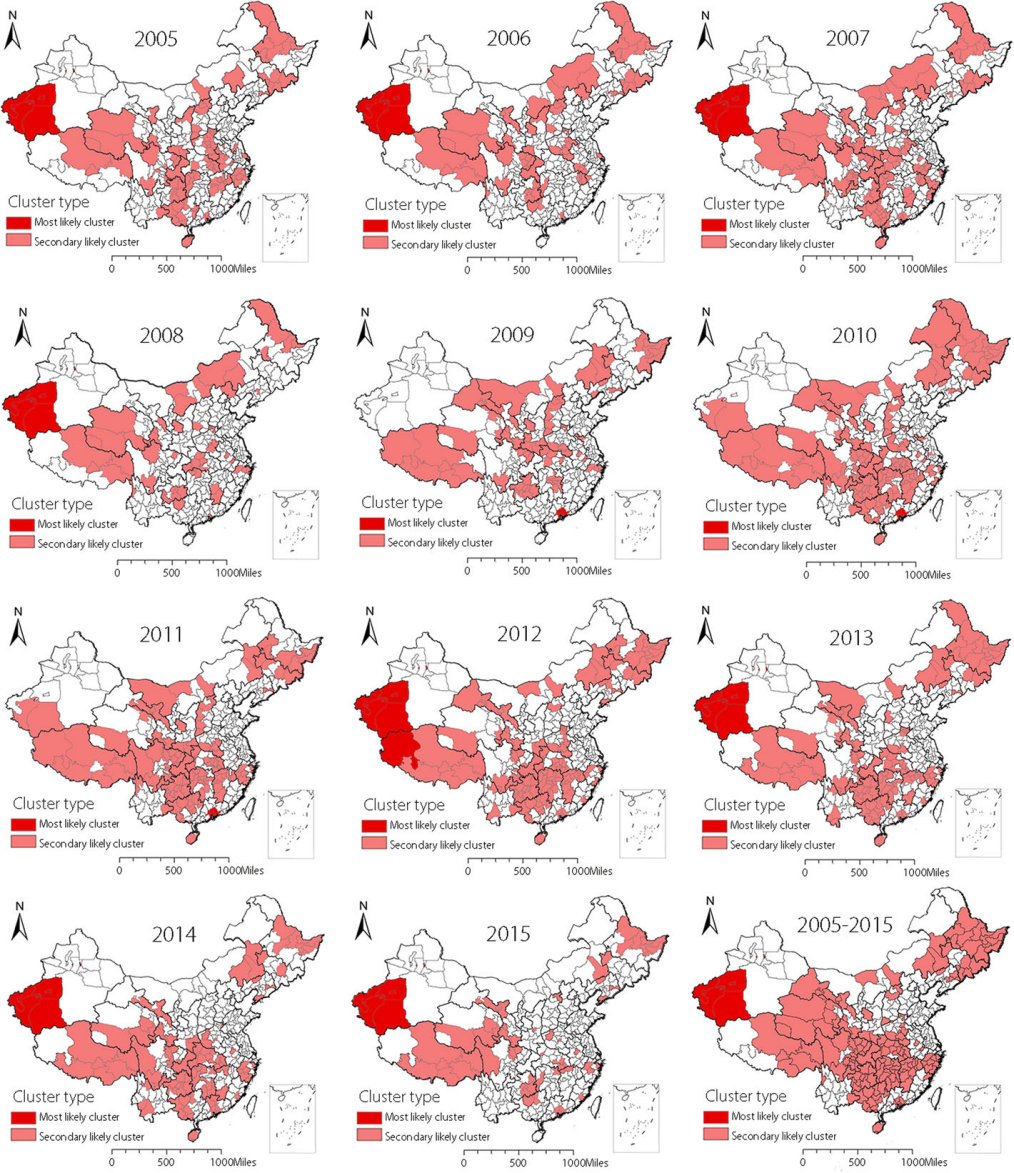
Spatial clustering of reported tuberculosis in the mainland of China from 2005 to 2015

Spatial clustering of the entire 11 years (2005–2015) identified a total of 166 prefectures were statistically significant high-risk areas with the spatial window covering 11% of the population at risk (Fig. 1). Spatial clustering analysis of every year from 2005 to 2015 found that the most likely clusters mainly distributed in two regions. One region was located in the southwest of Xinjiang Uygur Autonomous Region including Kashi Prefecture, Aksu Prefecture, Shihezi Prefecture, Hotan Prefecture and Kirgiz Autonomous Prefecture. The other region contained seven prefectures located in the southeast of Guangdong Province known as Guangzhou, Shenzhen, Zhuhai, Foshan, Huizhou, Dongguan and Zhongshan prefecture in the year of 2009, 2010 and 2011. The second likely clusters mainly scattered in some prefectures of northeastern China, western China and some central regions of China. Spatial clustering characteristics in every year from 2005 to 2015 were similar to the results in the total period with the most likely clusters distributed in the southwestern Xinjiang in most years. What’s more, the clustering areas presented a decreased tendency in recent years especially after 2010 (Fig. 1). The reported TB incidence in every year and the total research time emerged the same characteristics as the spatial clustering analysis (Additional file 3). That is to say, the high TB incidence prefectures were also mainly located in Xinjiang, Guangdong and so on. And the TB incidence presented a declining tendency. Spatial analysis at the prefecture level fixed the clustering location more clearly and more precisely crossing the provincial boundaries.

### Distribution of reported TB temporal clustering

**Fig. 2:**
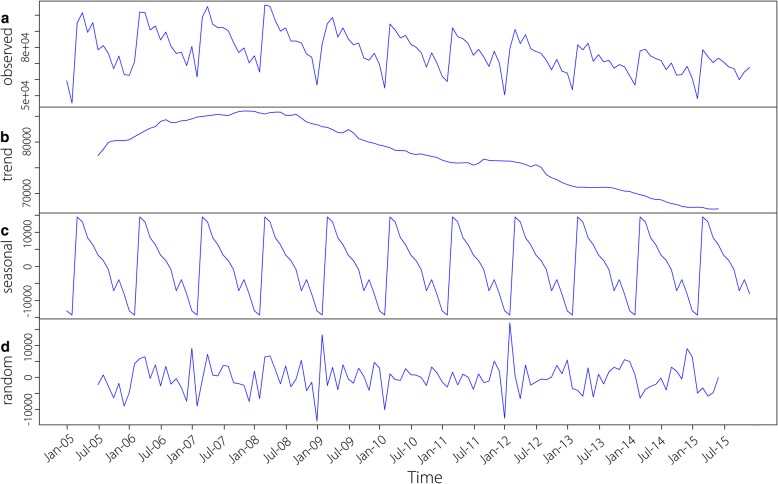
The seasonal distribution of monthly tuberculosis in the mainland of China from 2005 to 2015

The time series seasonal decomposition analysis of TB incidence showed a significant seasonal periodicity (Fig. 2). The temporal cluster analysis in every year showed the similar results to previous studies that TB incidence were concentrated in spring and early summer annually, ranging from February to June. The high aggregated time for TB in the whole study period was observed from March 2006 to May 2009. During this period, a total of 3 340 869 TB cases were reported, and the risk of TB related incidents was 19% (*RR* = 1.19, *P* <  0.001) higher than that in other time periods (Table 2). In addition, there was a declining trend for TB incidence generally in the study period but a slowly increase trend emerged from 2005 to 2007. (Fig. 2c).

**Table 2:** Temporal clustering of reported tuberculosis in the mainland of China from 2005 to 2015

Year	Cluster time frame	Observed cases	Expected cases	*RR*	*LLR*	*P*-value
2005	1 April–30 June 2005	286 531	231 097.37	1.35	8842.17	< 0.001
2006	1 March–31 May 2006	294 559	251 484.05	1.24	4760.61	< 0.001
2007	1 March–31 May 2007	298 483	258 417.27	1.22	4021.61	< 0.001
2008	1 March–31 May 2008	308 670	259 215.05	1.27	6062.04	< 0.001
2009	1 February–30 April 2009	275 579	239 579.59	1.21	3464.90	< 0.001
2010	1 March–31 May 2010	271 292	235 979.06	1.21	3424.59	< 0.001
2011	1 March–31 May 2011	264 335	229 929.34	1.21	3337.30	< 0.001
2012	1 March–31 May 2012	261 592	226 299.24	1.22	3559.64	< 0.001
2013	1 March–31 May 2013	242 821	215 559.30	1.18	2244.89	< 0.001
2014	1 March–31 May 2014	231 538	208 219.87	1.16	1704.83	< 0.001
2015	1 March–30 April 2015	152 886	134 380.27	1.17	1477.51	< 0.001
2005–2015	1 March 2006–31 May 2009	3 340 869	295 7242.48	1.19	34 214.95	< 0.001

*RR* Relative risk, *LLR* Log-likelihood ratios

### Distribution of reported TB spatio-temporal clustering

**Fig. 3:**
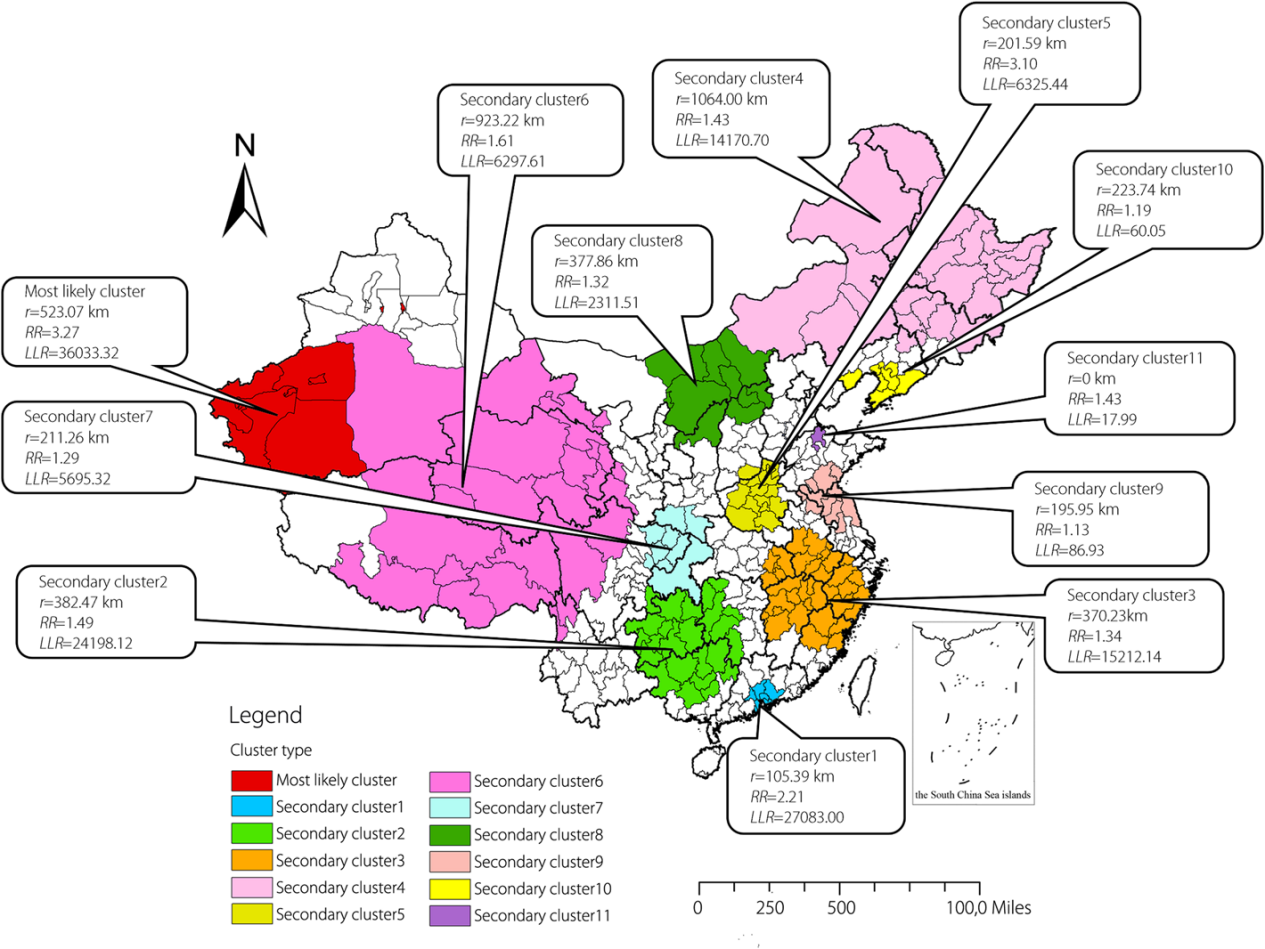
Spatio-temporal clustering of reported tuberculosis in the mainland of China from 2005 to 2015

The results of spatio-temporal cluster analysis for reported TB in 340 prefectures of mainland China from 2005 to 2015 were shown in Table 3 and Fig. 3. A most likely cluster area and eleven secondary cluster areas were detected in the study. The most likely spatio-temporal cluster area was located at the southwestern of Xinjiang Uygur Autonomous Region and the high-risk time was from September 2012 to October 2015 (*RR* = 3.27, *P* <  0.001). Besides, the area centered at Kirgiz Autonomous Prefecture (39.608093 N, 76.162029 E) with a radius of 523.07 km covered five prefectures just like the most likely cluster in the purely spatial analysis. Other eleven secondary clusters were mainly distributed in the western China, northeastern China and several relatively small areas in the center China. And the cluster time frames mainly ranged from March 2005 to June 2009 except the most likely cluster and secondary cluster1.

**Table 3:** Spatio-temporal clustering of reported tuberculosis in the mainland of China from 2005 to 2015

Cluster type	Cluster time frame	Coordinates/Radius	*N*	Observed cases	Expected cases	*RR*	*LLR*	*P*-value
Most likely cluster	1 September 2012–30 November 2015	(39.608093 N, 76.162029 E) / 523.07 km	5	73 552	22 572.11	3.27	36 033.32	< 0.001
Secondary cluster1	1 April 2009–30 June 2012	(22.898947 N, 113.887573 E) / 105.39 km	7	110 645	50 279.77	2.21	27 083.00	< 0.001
Secondary cluster2	1 April 2006–30 June 2009	(26.425756 N, 108.429245 E) / 382.47 km	24	351 481	238 013.99	1.49	24 198.12	< 0.001
Secondary cluster3	1 March 2005–31 May 2008	(28.754131 N, 117.356980 E) / 370.23 km	36	411 685	311 241.80	1.34	15 212.14	< 0.001
Secondary cluster4	1 March 2006–31 May 2009	(51.862772 N, 124.098025 E) / 1064.00 km	26	250 998	176 479.67	1.43	14 170.70	< 0.001
Secondary cluster5	1 June 2005–30 June 2005	(34.621634 N, 113.458645 E) / 201.59 km	15	13 933	4496.55	3.10	6325.44	< 0.001
Secondary cluster6	1 March 2006–31 May 2009	(33.910834 N, 93.584973 E) / 923.22 km	25	64 318	39 937.86	1.61	6297.61	< 0.001
Secondary cluster7	1 March 2005–31 May 2008	(31.993508 N, 107.064020 E) / 211.26 km	9	198 045	154 600.80	1.29	5695.32	< 0.001
Secondary cluster8	1 March 2005–31 May 2008	(41.492975 N, 110.349385 E) / 377.86 km	10	65 861	49 956.60	1.32	2311.51	< 0.001
Secondary cluster9	1 March 2005–31 May 2005	(34.554155 N, 119.103035 E) / 195.95 km	10	11 829	10 452.97	1.13	86.93	< 0.001
Secondary cluster10	1 March 2008–31 May 2008	(39.466843 N, 122.299835 E) / 223.74 km	7	4206	3534.86	1.19	60.05	< 0.001
Secondary cluster11	1 December 2005–31 December 2005	(37.483730 N, 117.822365 E) / 0 km	1	312	217.69	1.43	17.99	< 0.001

Most likely cluster: *P*-value < 0.001; Secondary cluster: *P*-value < 0.001

*N* number of prefectures in the cluster, *RR* Relative risk, *LLR* Log-likelihood ratios

### Distribution of SS+ reported TB and SS- reported TB spatio-temporal clustering

**Fig. 4:**
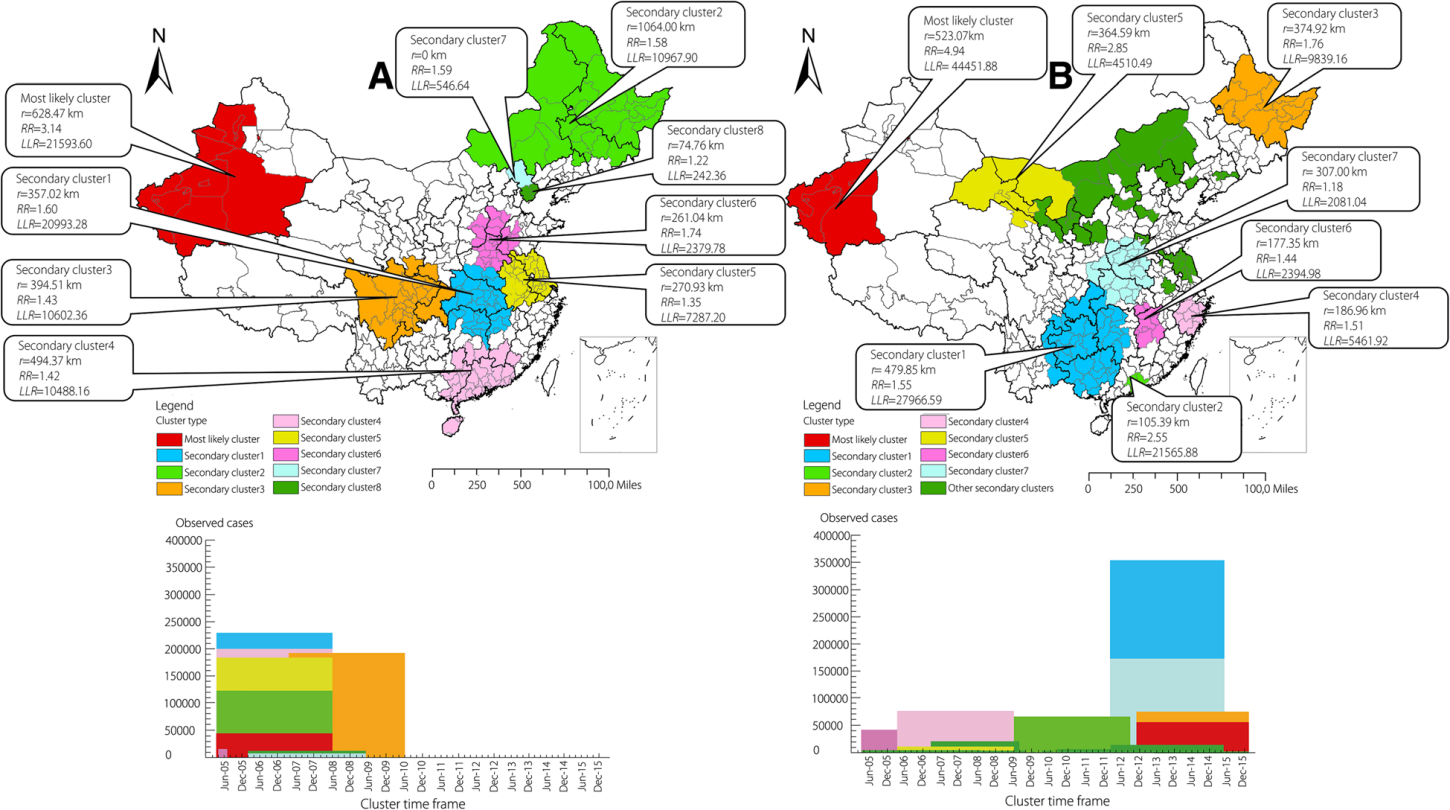
Spatio-temporal clustering of SS+ reported tuberculosis (**a**) and SS- reported tuberculosis (**b**) in the mainland of China from 2005 to 2015

Spatio-temporal clustering analysis showed that the most likely clusters of both SS+ reported TB and SS- reported TB were distributed in the southwestern of Xinjiang such as Kirgiz Autonomous Prefecture, Kashi Prefecture, Aksu Prefecture, Shihezi Prefecture and Hotan Prefecture, just like the clustering regions of total TB. But there were some differences in the distribution of secondary clusters between SS+ reported TB and SS- reported TB as shown in Fig. 4. In addition, the clustering time of SS+ reported TB was concentrated before 2010 while SS- reported TB was mainly concentrated after 2010. Additional files shows this in more detail (see Additional files 4 and 5).

## Discussion

Kulldorff’s retrospective scan statistics is one of the most powerful method to evaluate the spatial and temporal distribution of routinely collected data [11, 20] especially infectious diseases. Many countries have applied this method to tuberculosis research. A study from Canada used this method to reveal a complex coexistence of spatial and cohort clustering with the time of 1990 to 2013, and provided the basis for public health response [30]. Studies from Ghana [31] and American [32] about *Mycobacterium tuberculosis* strains or genotypes also found the spatio-temporal characteristics based on this method, which could guide the formulation of TB control and prevention policies. Though there were several studies in China have explored the distribution of TB, they were only restricted to a certain province or certain area, or the nationwide research just at the provincial level not the prefecture level. Research of a single province could find the specific clusters in that province but cannot get the clustering conditions in the whole country. And as for the nationwide research, we can get the clustering provinces in the whole country but cannot focus on a more specific area of the province. In this study, Geographic Information System (GIS) and Kulldorff’s scan statistical analysis were used to examine the changing patterns and clusters of TB between 2005 and 2015 in 340 prefectures in the mainland of China. The results detected a significantly spatial, temporal, and space-time clustering distribution of TB. Furthermore, spatio-temporal clusters of SS+ TB and SS- TB were also found in this research.

The spatial scanning results indicated that the spatial clusters existed in every year from 2005 to 2015 as well as the entire 11 years. The distribution of the clustering regions were similiar in each year, which meant the most likely clusters mainly included five prefectures of southwestern Xinjiang and seven prefectures of southeastern Guangdong. The main reasons of the high TB risk in five prefectures (Kashi, Aksu, Shihezi, Hotan and Kirgiz Autonomous Prefecture) of Xinjang are relatively poor economy, high proportion of minorities and underdeveloped medical level [33, 34]. It was no doubt that Guangdong is a developed and prosperous province, but the immigrants accounts for a large part of the population especially in the seven prefectures (Guangzhou, Shenzhen, Zhuhai, Foshan, Huizhou, Dongguan and Zhongshan). For example, approximately three fourths population in Shenzhen are immigrants and the reported TB incidence in Shenzhen are 381.3 per 100 000 from 2006 to 2010. Immigrants maybe one of the most important factors of the high TB incidence in Gungdong Province [35]. The secondary clusters were scattered in almost every province in every year, but both the number of clusters and the reported TB incidences showed a downward trend in recent years owing to the effective prevention and control measures. In short, the five prefectures in Xinjiang and seven prefectures in Guangdong remain the key areas for TB prevention and control in the future. Other clustering areas also should take appropriate measures corresponding to the specific reasons to control TB, and achieve the “End TB Strategy” in China earlier.

The temporal scanning results displayed the high risk month of TB in every year from 2005 to 2015 and the entire 11 years. The epidemic season of TB in the mainland of China for each year were basically the same, which mainly concentrated in the spring and the early summer between February to June. This result was consistent with other previous studies in the United States [28], northern India [36], Wuhan City [26] and Yunnan Province [10] in China. In winter, the reduction on exposure to ultraviolet from sunlight and the deficiency of vitamin D, the increase of serious pollution days, more time in indoor activities with poor ventilation [37, 38], all of those could raise the risk of TB infection. Additionally, the Chinese traditional festival called Spring Festival is another special reason that causes the delaying health-seeking. It usually takes some weeks even several month from TB infection to symptom onset and diagnosis [39]. That’s why the temporal clustering time were in spring and early summer. Of course we should keep a close eye on the peak period of TB so as to reduce the TB incidence in future. What’s more, the long term trend of reported TB in 2005–2015 showed a declining trendency on the whole, which indicated the achievements of TB have been made in recent years.

Spatio-temporal cluster analysis identified five prefectures in the southwestern of Xinjiang Uygur Autonomous Region from September 2012 to October 2015 were the most likely cluster. As a matter of fact, every year’s TB incidences in the southwestern of Xinjiang were very high. The clustering time were concentrated in September 2012 to October 2015, which indicated that prevention and control measures in this region still need to be strengthened not only in the distant past, but also in recent years. Other secondary clusters distributed in the western China, northeastern China and scattered in the center China mainly from March 2005 to June 2009, which meant these clusters were once the high TB incidence regions but took a favourable turn in recent years. The spatio-temporal cluster analysis about SS+ reported TB and SS- reported TB also found that prefectures in the southwestern of Xinjiang were the most likely clusters which was the same as the results of all the TB cases analysis, but the clustering time of SS+ reported TB was from 2005 to 2010 while the clustering time of SS- reported TB was mainly from 2010 to 2015. Although prevention and control of TB, such as directly observed treatment strategy (DOTS) have made great progress, we still need to attach much importance to both SS+ TB and SS- TB. As for the strong infectious SS+ TB, the clustering results could guide us to prevent and discover the outbreak and epidemic of TB. The TB incubation period of children is short, so if known the aggregation characteristics of SS+ TB, we located the clustering time and space, and make early corresponding measures. As for the SS- TB, though it doesn’t have the strong infectivity, it has great potential hazards. Moreover, SS- TB is difficult to diagnose because no bacteria can be seen in the smear and on L-J medium. So one of the most challenging questions is how to improve the diagnostic sensitivity and accuracy of SS- TB. There are many new techniques have arisen in recent years due to the development of bio-sensing techniques and the device platform for TB detection. Advancements in transduction and nanotechnology in biosensors such as optical detection techniques and electrochemical detection techniques could improve the sensitivity and specificity in detecting biomarkers in complex sample matrices (urine, serum, saliva, blood) using little amount of sample, and show remarkable features in TB diagnosis compared to the conventional techniques [40]. The development of the device platform including non-molecular techniques and molecular techniques such as interferon-gamma (IFN-γ) release assays (IGRA), loop-mediated isothermal amplification (LAMP), GeneXpert analyser are emerged to ensure the fulfillment of unmet requirements in TB diagnostics such as rapidity, affordability, simplicity, precision and high sensitivity [41]. With the development of new technology, more methods will be used to diagnose SS- TB.

This study was subject to some limitations. First, we conducted spatio-temporal scan statistic to detect clusters in different space and periods of time but this method only relies on circular spatial scanning and cylinder space-time scanning, and doesn’t allow for irregular space. Second, other ecological or individual level factors affected TB incidence such as low economic level [42], poor living environment [43], backward educational and medical conditions [34], exposure to ambient air pollution [38], age, sex, smoking and so on have not been taken into account in the present study. Third, the TB risk may be underestimated because of the missing cases’ report in some areas and the prefecture is not the smallest unit of administrative regionalization.

## Conclusions

This study identified significant spatial, temporal and spatio-temporal clusters of reported TB cases at the prefecture level in the mainland of China from 2005 to 2015. The southwest of Xinjiang Uygur Autonomous Region and the southeast of Guangdong Province were the most likely clustering areas while the spring and early summer were the most likely clustering time. The spatio-temporal clustering results showed that the clustering time of SS+ TB was concentrated before 2010 while SS- TB was mainly concentrated after 2010. In a word, our result is helpful in prioritizing resource assignment in high-risk periods and high-risk areas, and to formulate powerful strategy to prevention and control TB.

## Additional files

Additional file 1: Multilingual abstract in the six official working languages of the United Nations. (PDF 430 kb)

Additional file 2: The name, area and geographical position of 340 prefectures in the mainland of China (DOCX 755 kb)

Additional file 3: The reported tuberculosis incidence of 340 prefectures in the mainland of China from 2005 to 2015 (TIF 15157 kb)

Additional file 4: Spatio-temporal clustering of sputum smear-positive tuberculosis in the mainland of China from 2005 to 2015. (DOCX 16 kb)

Additional file 5: Spatio-temporal clustering of sputum smear-negative tuberculosis in the mainland of China from 2005 to 2015. (DOCX 19 kb)

## Abbreviations

CISDCP: China Information System for Disease Control and Prevention
TB: Tuberculosis
SS +: Sputum smear-positive
SS-: Sputum smear-negative
WHO: World Health Organization
*RR*: Relative risk
*LLR*: Log-likelihood ratios
GIS: Geographic Information System
DOTS: Directly observed treatment strategy
HIV: Human immunodeficiency virus
IGRA: Interferon-gamma (IFN-γ) release assays
LAMP: Loop-mediated isothermal amplification
