# Supplementary material for: Spatial and temporal clustering analysis of tuberculosis in the mainland of China at the prefecture level, 2005–2015
Source: Infect Dis Poverty. 2018 Oct 20;7:106. doi: 10.1186/s40249-018-0490-8 (PMC6195697; doi:10.1186/s40249-018-0490-8)
Supplement: Supplementary file 1 — Multilingual abstract in the six official working languages of the United Nations. (PDF 430 kb) [file 40249_2018_490_MOESM1_ESM.pdf]

Translation of the abstract into the six official working languages of the United Nations

تحليل التكتل الزماني والمكاني لمرض السل في مقاطعات بر الصين الرئيسي لعام 2005-2015

منغ-يانغ ليو، كي-هوان لي، يينغ-جي زانغ، يوان ما، يو ليو، وي فينغ، شينغ-بي هو، إنداووك أمسالو، شيا لي، وي وانغ، وي-مين لي، شيو-هوا غوو

المُلَخَّصّ

الخلفية: ما زال داء السل (TB) أحد أخطر الأمراض المعدية في بر الصين الرئيسي. لذلك كان من الملح وضع تدابير أكثر فعالية لمكافحته والوقاية منه.

المنهجية:حصلنا على البيانات الخاصة بحالات داء السل في 340 محافظة من محافظات بر الصين الرئيسي تم الإبلاغ عنها في الفترة من يناير 2005 إلى ديسمبر 2015 من نظام معلومات الصين لمكافحة الأمراض والوقاية منها (CISDCP). كما استعنّا بإحصائيات Kulldorff للمسح الزماني والمكاني بأثر رجعي لتحديد التكتلات الزمانية والمكانية والمكانية الزمانية الخاصة بالسل المبلغ عنها في بر الصين الرئيسي باستخدام نموذج بواسون المنفصل للاحتمالات. واكتشفنا وجود تكتلات زمانية مكانية لحالات السل المبلغ عنها في جميع المقاطعات، بنوعيها إيجابي لطخة البصاق (SS+) وسلبي لطخة البصاق (SS-)

النتائج: بلغ مجموع حالات السل المبلغ عنها 10,200,528 حالة تم جمعها من 340 مقاطعة في الفترة من العام 2005 إلى العام 2015، من بينها 5,283,983 حالة سلبية اللطخة و 4,631,734 حالة إيجابية اللطخة بنتائج واضحة لاختبار لطخة البصاق، و284,811 حالة لم تخضع لاختبار لطخة البصاق. تجدر الإشارة إلى أنه قد لوحظ في هذا البحث وجود أنماط للتكتلات المكانية والزمانية والزمانية المكانية لمرض السل. إذ خلصت نتائج مسح Kulldorff إلى وجود اثنتي عشر تكتل زماني مكاني ضخم لحالات السل المبلغ عنها كان التكتل الزماني المكاني الأرجح (نسبة الاختطار = 3.27, القيمة الاحتمالية < 0.001) موجود بالدرجة الأولى في منطقة سنجان ذاتية الحكم غربيّ الصين ويغطي خمس مقاطعات وتكتلات خلال الإطار الزمني ما بين سبتمبر 2012 ونوفمبر 2015. كما أظهرت نتائج التكتلات الزمانية المكانية لحالات السل إيجابية لطخة البصاق وسلبية لطخة البصاق كون التكتلات المرجحة موزعة في غربيّ الصين. ومع ذلك ، تركز تكتل حالات السل إيجابية لطخة البصاق زمنيًا قبل عام 2010 بينما تركز تكتل حالات السل سلبية لطخة البصاق زمنيًا بالدرجة الأولى بعد عام 2010.

الاستنتاجات: حددت هذه الدراسة بسهولة زمان ومكان تكتل حالات السل إيجابية لطخة البصاق وسلبية لطخة البصاق في 340 مقاطعة من مقاطعات بر الصين الرئيسي، مما من شأنه أن يساعد في تحديد أولويات تخصيص الموارد في الفترات والمناطق مرتفعة الخطورة، وكذلك في إعداد استراتيجية قوية للوقاية من السل ومكافحته.

Translated from English version into Arabic by Malika 2012, proofread by Heba Kandel, through

TRANSLATORS
WITHOUT BORDERS

2005–2015 年中国大陆地市级水平结核病时空聚类分析

刘梦阳，李琪欢，张英杰，马圆，刘悦，冯巍，侯城北，Endawoke Amsalu，李霞，王嵬，李卫民，郭秀花

摘要

引言：结核病（TB）仍是中国大陆最严重的传染病之一。因此，制定更有效的结核病防治措施是十分迫切的。

方法：从中国疾病预防控制中心信息系统收集 2005 年 1 月−2015 年 12 月中国大陆 340 个地级市的报告结核病数据。基于离散的泊松概率模型，利用 Kulldorff 回顾性时空扫描统计方法，探测中国大陆报告结核病的时间、空间和时空聚类特征。本研究也分析了痰涂片阳性（SS+）和痰涂片阴性（SS-）报告结核在地市级水平上的时空聚类特点。
结果：本研究共收集 2005−2015 年 340 个地级市报告结核病病例 10,200,528 例，其中包括有明确涂片检查结果的 SS+ TB 5,283,983 例，SS- TB 4,631,734 例，以及没有涂片结果的 TB 284,811 例。本研究发现结核发病在空间、时间和时空分布上具有显著的聚集特点。Kulldorff 扫描结果发现 12 个区域出现报告结核病的显著时空聚集。第一时空聚类区（RR=3.27，*P*<0.001）主要位于中国西部的新疆维吾尔自治区内，覆盖了 5 个地级市，且聚集时间为 2012 年 9 月−2015 年 11 月。SS+ TB 和 SS- TB 的时空聚类结果同样显示第一聚类区分布在中国西部。但是，SS+ TB 的聚集时间集中在 2010 年之前，而 SS- TB 主要集中在 2010 年之后。
结论：本研究识别了中国大陆 340 个地级市 TB、SS+ TB 和 SS- TB 容易聚集的时间和地区，为在高危时期和高危地区优先进行资源配置，以及制定有效的结核病防治策略提供了帮助。

Translated from English version into Chinese by Meng-Yang Liu

Analyse d'agrégats spatio-temporels de cas de tuberculose au niveau préfectoral de la Chine continentale, 2005-2015

Meng-Yang Liu, Qi-Huan Li, Ying-Jie Zhang, Yuan Ma, Yue Liu, Wei Feng, Cheng-Bei Hou, Endawoke Amsalu, Xia Li, Wei Wang, Wei-Min Li, Xiu-Hua Guo

Résumé

Contexte: La tuberculose reste toujours l'une des maladies infectieuses les plus graves en Chine continentale. Il était donc urgent d'élaborer des mesures plus efficaces en vue de la prévenir et la contrôler.
Méthodes: Les données des cas de tuberculose déclarés dans 340 préfectures de la Chine continentale ont été obtenues à partir du système d'information chinois pour le contrôle et la prévention des maladies (CISDCP), entre janvier 2005 et décembre 2015. La statistique de balayage spatio-temporel rétrospectif de Kulldorff a permis d'identifier les agrégats temporels, spatiaux et spatio-temporels de cas de tuberculose déclarés en Chine continentale, à l'aide du modèle de probabilité discrète de la loi de Poisson. Des agrégats spatio-temporels de tuberculose à frottis positif (SS+) et de tuberculose à frottis négatif (SS-) ont également été détectés au niveau de la préfecture.
Résultats: Au total, 10 200 528 cas de tuberculose ont été recensés entre 2005 et 2015 dans 340 préfectures, dont 5 283 983 cas de tuberculose SS- et 4 631 734 cas de tuberculose SS+, suite aux examens spécifiques de frottis d'expectoration, et 284 811 cas sans examen de frottis d'expectoration. Cette recherche a permis d'observer une tendance significative d'agrégats de cas de tuberculose au niveau spatial, temporel et spatio-temporel. Les résultats de la statistique de balayage de Kulldorff ont révélé douze agrégats spatio-temporels significatifs de cas déclarés de tuberculose. L'agrégat spatio-temporel le plus représentatif (RR = 3,27, P < 0,001) était principalement situé dans la région autonome de Xinjiang Uygur, en Chine occidentale, et concernait cinq préfectures, entre septembre 2012 et novembre 2015. Les résultats des agrégations spatio-temporelles de tuberculose à SS+ et SS- ont également montré les agrégats les plus représentatifs en Chine occidentale. Cependant, la période d'agrégation de la tuberculose

SS+ était concentrée avant 2010, alors que la tuberculose SS- était principalement concentrée après 2010.
Conclusions: La présente étude a permis d'identifier la période et la région d'agrégation de la tuberculose à SS+ et SS-, dans 340 préfectures de la Chine continentale. Ces résultats sont utiles pour établir l'ordre de répartition des ressources pendant les périodes à risque élevé et dans les zones à risque élevé et pour élaborer une stratégie efficace de prévention et de contrôle de la tuberculose.

Translated from English version into French by Agnès Giner, proofread by Chrystelle C. Ngateu, through

TRANSLATORS
WITHOUT BORDERS

Пространственно-временной кластерный анализ туберкулеза в континентальной части Китая на уровне префектур, 2005–2015 гг.

Мэн-Ян Лю, Ци-Хуан Ли, Ин-Цзе Чжан, Юань Ма, Юэ Лю, Вэй Фэн, Чэн-Бэй Хоу, Эндавоке Амсалу, Ся Ли, Вэй Ван, Вэй-Минь Ли, Сю-Хуа Го

Краткий обзор

Справочная информация: Туберкулез (ТБ) остается одним из самых серьезных инфекционных заболеваний в континентальной части Китая. По этой причине требовалась срочная разработка более эффективных мер по его предотвращению и контролю.
Методы: Данные о зарегистрированных случаях ТБ в 340 префектурах континентальной части Китая были получены от Китайского центра по контролю и профилактике заболеваний с января 2005 по декабрь 2015 гг. С помощью ретроспективного пространственно-временного статистического сканирования Куллдорфа были выявлены пространственные, временные и пространственно-временные кластеры зарегистрированного ТБ в континентальной части Китая с использованием распределения Пуассона (вероятностного распределения дискретного типа). Также на уровне префектур были выявлены пространственно-временные кластеры случаев зарегистрированного туберкулеза в открытой (с выделением бактерий, БК+) и закрытой (без бактериовыделения, БК-) формах.
Результаты: Всего было собрано 10 200 528 зарегистрированных случаев туберкулеза в период с 2005 по 2015 гг. в 340 префектурах. Среди них — 5 283 983 случаев ТБ в закрытой форме и 4 631 734 в открытой форме при наличии результатов исследования мокроты, а также 284 811 случаев без исследования мокроты. Важно отметить, что в данном исследовании были отмечены кластерные паттерны ТБ в пространстве, времени и пространстве-времени. По результатам сканирования Куллдорфа было обнаружено 12 статистически значимых пространственно-временных кластеров зарегистрированного ТБ. Наиболее вероятный пространственно-временной кластер (*ОР* = 3,27, p < 0,001) находился главным образом в Синьцзян-Уйгурском автономном районе на западе Китая, охватывал пять префектур и кластеризовался во временном промежутке с сентября 2012 до ноября 2015 гг. Результаты пространственно-временной кластеризации ТБ в открытой и закрытой форме также показали наиболее вероятные кластеры, распределенные в

западном Китае. Однако время кластеризации ТБ в открытой форме было сосредоточено до 2010 года, а ТБ в закрытой форме — после 2010 года.
Выводы: В данном исследовании были определены зоны и временные периоды ТБ, ТБ в открытой форме и ТБ в закрытой форме, которые четко делились на кластеры в 340 префектурах континентальной части Китая. Это полезно для определения приоритетности назначения ресурсов в зоны и периоды высокого риска, а также для разработки эффективной стратегии контроля и профилактики ТБ.

Translated from English version into Russian by Polina Nikitina, proofread by Sam Abramov, through

TRANSLATORS
WITHOUT BORDERS

Análisis de concentración espacial y temporal de la tuberculosis en la China continental por prefecturas, 2005-2015

Meng-Yang Liu, Qi-Huan Li, Ying-Jie Zhang, Yuan Ma, Yue Liu, Wei Feng, Cheng-Bei Hou, Endawoke Amsalu, Xia Li, Wei Wang, Wei-Min Li, Xiu-Hua Guo

Resumen
Antecedentes: La tuberculosis (TB) todavía es una de las enfermedades contagiosas más graves en la China continental. Por tanto, era urgente formular medidas más efectivas para prevenirla y controlarla.
Métodos: Los datos de casos notificados de TB en 340 prefecturas de la China continental se obtuvieron a partir del Sistema de Información de China para el Control y la Prevención de Enfermedades entre enero de 2005 y diciembre de 2015. Se utilizaron las estadísticas retrospectivas de barrido en espacio-tiempo de Kulldorff para identificar los grupos temporales, espaciales y espacio-temporales de los casos notificados de TB en la China continental aplicando el modelo discreto de probabilidad de Poisson. También se detectaron grupos espacio-temporales de TB notificada con frotis de esputo positivo (SS+) y TB notificada con frotis de esputo negativo (SS-) por prefecturas.
Resultados: Se recogieron un total de 10.200.528 casos notificados de TB entre 2005 y 2015 en 340 prefecturas, incluyendo 5.283.983 casos de TB SS- y 4.631.734 casos de TB SS+ con resultados específicos de frotis de esputo, así como 284.811 casos sin pruebas de frotis de esputo. Como resultado de esta investigación se observaron patrones significativos de concentración espacial, temporal y espacio-temporal de TB. Los resultados de escaneo de Kulldorff hallaron doce grupos espacio-temporales significativos de TB notificada. El grupo espacio-temporal más probable (RR=3,27, P < 0,001) se localizó principalmente en la región autónoma Uygur de Xinjiang del oeste de China, abarcando cinco prefecturas y concentradao entre septiembre de 2012 y noviembre de 2015. Los resultados del grupo espacio-temporal de TB SS+ y TB SS- también mostraron que las concentraciones más probables se distribuían por el oeste de China. Sin embargo, el tiempo de concentración de SS + TB se produjo principalmente antes de 2010, mientras que SS-TB se concentró principalmente después de 2010.
Conclusiones: Este estudio ha identificado el tiempo y la región de TB, TB SS+ y TB SS- concentrados

en 340 prefecturas de la China continental, lo cual resulta útil para priorizar la asignación de recursos en períodos y áreas de alto riesgo, así como para elaborar una potente estrategia destinada a la prevención y control de TB.

Translated from English version into Spanish by Rebeca Barrero Medina, proofread by SergioLorenzi, through

TRANSLATORS
WITHOUT BORDERS
