# Supplementary material for: Spatial and temporal clustering analysis of tuberculosis in the mainland of China at the prefecture level, 2005–2015
Source: Infect Dis Poverty. 2018 Oct 20;7:106. doi: 10.1186/s40249-018-0490-8 (PMC6195697; doi:10.1186/s40249-018-0490-8)
Supplement: Supplementary file 2 — The name, area and geographical position of 340 prefectures in the mainland of China (DOCX 755 kb) [file 40249_2018_490_MOESM2_ESM.docx]

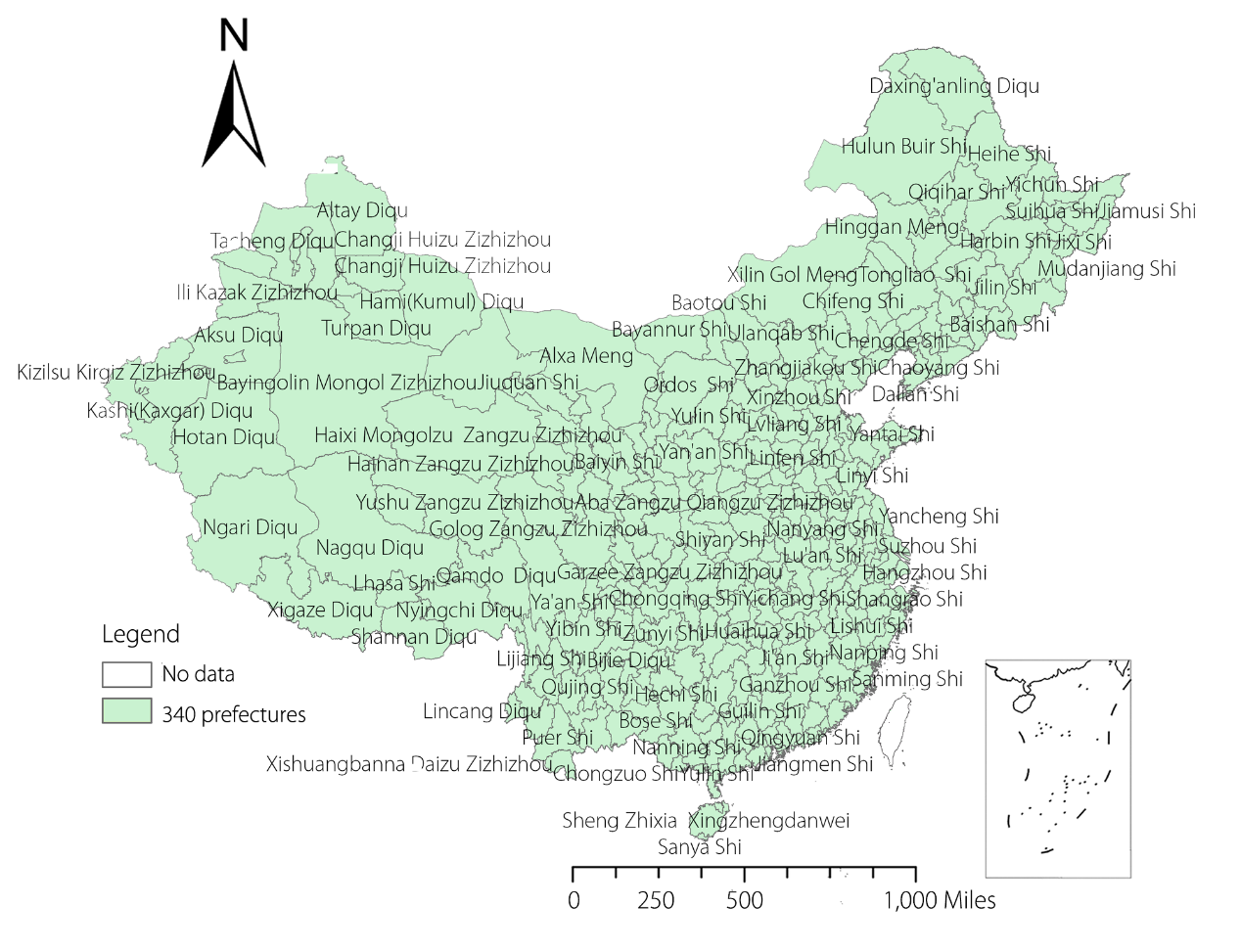


The presentation of 340 prefectures in the mainland of China.

(But not all the name of the prefectures were shown in the map.)

Additional Table S1. The name, area and geographical position of 340 prefecture in the mainland of China.

| FID | NAME | AREA | LONGITUDE | LATITUDE |
| --- | --- | --- | --- | --- |
| 1 | Shijiazhuang Shi | 14046.77421 | 114.494145 | 38.09842299 |
| 2 | Qinhuangdao Shi | 7749.354496 | 119.20795 | 40.0216425 |
| 3 | Handan Shi | 12054.6285 | 114.467905 | 36.53043549 |
| 4 | Xingtai Shi | 12474.24246 | 114.800655 | 37.27215801 |
| 5 | Baoding Shi | 22278.0391 | 115.040325 | 39.099945 |
| 6 | Zhangjiakou Shi | 36651.06018 | 115.13526 | 40.8623865 |
| 7 | Chengde Shi | 39434.20601 | 117.56951 | 41.400669 |
| 8 | Cangzhou Shi | 14103.04062 | 116.75081 | 38.20680048 |
| 9 | Langfang Shi | 6409.528752 | 116.52312 | 39.05278749 |
| 10 | Hengshui Shi | 8824.90304 | 115.869995 | 37.70976048 |
| 11 | Taiyuan Shi | 6968.471653 | 112.32813 | 37.93716399 |
| 12 | Datong Shi | 14010.71255 | 113.555935 | 39.8915445 |
| 13 | Yangquan Shi | 4570.977431 | 113.4755 | 38.0917875 |
| 14 | Changzhi Shi | 13976.97485 | 112.851335 | 36.47568501 |
| 15 | Shuozhou Shi | 10659.02038 | 112.728865 | 39.6916275 |
| 16 | Xinzhou Shi | 25111.20463 | 112.45027 | 38.89655298 |
| 17 | Lvliang Shi | 21015.58166 | 111.336875 | 37.73466501 |
| 18 | Jinzhong Shi | 16361.3144 | 112.76755 | 37.3711545 |
| 19 | Dalian Shi | 12779.01398 | 122.299835 | 39.46684251 |
| 20 | Benxi Shi | 8439.507514 | 124.682045 | 41.19520749 |
| 21 | Dandong Shi | 14653.09361 | 124.53773 | 40.45676799 |
| 22 | Yingkou Shi | 5186.117885 | 122.480465 | 40.43669298 |
| 23 | Fuxin Shi | 10391.01667 | 121.99136 | 42.27491349 |
| 24 | Panjin Shi | 3263.350295 | 122.02251 | 41.06102949 |
| 25 | Chaoyang Shi | 19658.79045 | 120.0644 | 41.49120699 |
| 26 | Changchun Shi | 20461.93618 | 125.80994 | 44.25328248 |
| 27 | Jilin Shi | 27460.38741 | 126.77602 | 43.60237899 |
| 28 | Siping Shi | 14332.59908 | 124.54562 | 43.485369 |
| 29 | Liaoyuan Shi | 5151.072164 | 125.332795 | 42.76274898 |
| 30 | Tonghua Shi | 15528.16843 | 125.99667 | 41.94039699 |
| 31 | Baishan Shi | 17415.52839 | 127.21039 | 42.090351 |
| 32 | Songyuan Shi | 21153.031 | 124.63954 | 44.7627795 |
| 33 | Baicheng Shi | 25627.98443 | 122.992545 | 45.26289549 |
| 34 | Yanbian Chaoxianzu Zizhizhou | 43327.3219 | 129.38889 | 43.25092299 |
| 35 | Harbin Shi | 53034.23883 | 127.95785 | 45.36744699 |
| 36 | Qiqihar Shi | 42083.60138 | 124.523205 | 47.56958001 |
| 37 | Jixi Shi | 22346.57775 | 132.1599 | 45.7301295 |
| 38 | Hegang Shi | 14383.20675 | 132.425402 | 47.7078435 |
| 39 | Daqing Shi | 21134.4391 | 124.769385 | 46.42796349 |
| 40 | Yichun Shi | 32527.50193 | 129.199285 | 47.95279098 |
| 41 | Qitaihe Shi | 6212.874804 | 131.00521 | 45.954819 |
| 42 | Mudanjiang Shi | 38540.96947 | 129.76155 | 44.70022401 |
| 43 | Heihe Shi | 66395.10669 | 127.12613 | 49.29272448 |
| 44 | Suihua Shi | 34817.51215 | 126.711665 | 46.7949735 |
| 45 | Wuxi Shi | 4647.005867 | 120.324474 | 31.5648585 |
| 46 | Changzhou Shi | 4352.106572 | 119.8790022 | 31.61029701 |
| 47 | Nantong Shi | 8577.005474 | 121.0482 | 32.19786399 |
| 48 | Lianyungang Shi | 7405.004975 | 119.103035 | 34.55415501 |
| 49 | Huai'an Shi | 9996.60441 | 118.927025 | 33.40724751 |
| 50 | Yancheng Shi | 14916.84286 | 120.18419 | 33.5285055 |
| 51 | Taizhou Shi | 5788.546602 | 120.093735 | 32.57771598 |
| 52 | Suqian Shi | 8581.736213 | 118.5527 | 33.7827375 |
| 53 | Huludao Shi | 10316.22323 | 120.1237 | 40.59720648 |
| 54 | Yangzhou Shi | 6613.368166 | 119.455275 | 32.815353 |
| 55 | Zhenjiang Shi | 3825.6846 | 119.464145 | 31.97694399 |
| 56 | Tieling Shi | 12957.5616 | 124.27412 | 42.73767699 |
| 57 | Fushun Shi | 11250.48569 | 124.567635 | 41.85782799 |
| 58 | Shuangyashan Shi | 21974.29512 | 132.498 | 46.68352698 |
| 59 | Jiamusi Shi | 31907.90865 | 132.286545 | 47.19627348 |
| 60 | Anshan Shi | 9175.697247 | 122.96873 | 40.78278351 |
| 61 | Liaoyang Shi | 4668.336456 | 123.131015 | 41.15628048 |
| 62 | Shenyang Shi | 12806.89689 | 123.11056 | 42.11532399 |
| 63 | Jinzhou Shi | 9724.571894 | 121.6606534 | 41.47583949 |
| 64 | Yuncheng Shi | 13996.80617 | 111.158655 | 35.20444899 |
| 65 | Tangshan Shi | 13133.53654 | 118.40841 | 39.74552499 |
| 66 | Linfen Shi | 20297.85011 | 111.47194 | 36.162342 |
| 67 | Jincheng Shi | 9420.737263 | 112.77712 | 35.633274 |
| 68 | Beijing Shi | 16369.62347 | 116.456505 | 40.250631 |
| 69 | Tianjin Shi | 11493.78702 | 117.38399 | 39.40713099 |
| 70 | Shanghai Shi | 6259.559046 | 121.380405 | 31.106247 |
| 71 | Daxing'anling Diqu | 81697.5632 | 124.098025 | 51.86277198 |
| 72 | Xuzhou Shi | 11239.67051 | 117.512545 | 34.33166901 |
| 73 | Nanjing Shi | 6594.658694 | 118.79825 | 31.9209405 |
| 74 | Suzhou Shi | 8469.59295 | 120.622415 | 31.3990335 |
| 75 | Baotou Shi | 27701.01322 | 110.349385 | 41.49297501 |
| 76 | Chifeng Shi | 85057.71172 | 118.700585 | 43.24220451 |
| 77 | Hinggan Meng | 54815.83372 | 121.55081 | 45.95598798 |
| 78 | Tongliao Shi | 58812.94624 | 121.474875 | 43.941744 |
| 79 | Xilin Gol Meng | 200874.4116 | 115.52576 | 44.177277 |
| 80 | Bayannur Shi | 64318.60748 | 107.55309 | 41.3178675 |
| 81 | Zhuzhou Shi | 11284.08109 | 113.532935 | 27.0348465 |
| 82 | Meizhou Shi | 15839.61831 | 116.12549 | 24.15572652 |
| 83 | Shanwei Shi | 4815.276901 | 115.50377 | 23.06562549 |
| 84 | Yangjiang Shi | 7805.227985 | 111.823365 | 22.09213251 |
| 85 | Zhongshan Shi | 1638.919854 | 113.369205 | 22.48650702 |
| 86 | Chaozhou Shi | 3117.253239 | 116.77555 | 23.8286745 |
| 87 | Yunfu Shi | 7805.406748 | 111.78588 | 22.85563401 |
| 88 | Guilin Shi | 27702.06319 | 110.548075 | 25.32261549 |
| 89 | Guigang Shi | 10647.50596 | 109.92766 | 23.3564205 |
| 90 | Hechi Shi | 33491.42032 | 107.864385 | 24.59299299 |
| 91 | Sanya Shi | 1844.050626 | 109.388615 | 18.39023652 |
| 92 | Mianyang Shi | 20245.35158 | 104.73085 | 31.87372899 |
| 93 | Guangyuan Shi | 16225.55528 | 105.686575 | 32.23145349 |
| 94 | Suining Shi | 5330.009472 | 105.525515 | 30.673356 |
| 95 | Leshan Shi | 12892.9008 | 103.575895 | 29.17224801 |
| 96 | Yibin Shi | 13282.71484 | 104.47704 | 28.55931999 |
| 97 | Dazhou Shi | 16558.82808 | 107.595505 | 31.33485048 |
| 98 | Bazhong Shi | 12314.48754 | 107.06402 | 31.99350849 |
| 99 | Qianxi'nan Buyizu Miaozu Zizhizhou | 16834.06991 | 105.53362 | 25.39943052 |
| 100 | Anshun Shi | 9335.196839 | 105.90585 | 25.98995502 |
| 101 | Weinan Shi | 13153.21821 | 109.78152 | 35.03060349 |
| 102 | Yan'an Shi | 36939.01368 | 109.102425 | 36.42676749 |
| 103 | Hanzhong Shi | 27144.30085 | 106.883955 | 33.01347951 |
| 104 | Ankang Shi | 23408.11031 | 109.114965 | 32.777505 |
| 105 | Shangluo Shi | 19629.58517 | 109.79455 | 33.766905 |
| 106 | Jiayuguan Shi | 1298.842315 | 98.16408901 | 39.81377001 |
| 107 | Jinchang Shi | 7382.981764 | 102.04938 | 38.51307849 |
| 108 | Longnan Shi | 28014.73249 | 105.3063 | 33.56150598 |
| 109 | Pingliang Shi | 11070.23112 | 106.599255 | 35.3149755 |
| 110 | Qingyang Shi | 27163.76498 | 107.534275 | 36.19742949 |
| 111 | Linxia Huizu Zizhizhou | 8079.723224 | 103.268385 | 35.5787715 |
| 112 | Wuzhou Shi | 12580.94358 | 110.989775 | 23.49593649 |
| 113 | Haidong Diqu | 13009.56084 | 101.98027 | 36.30323049 |
| 114 | Xining Shi | 7215.12172 | 101.414875 | 36.82294248 |
| 115 | Haikou Shi | 2179.05085 | 110.41889 | 19.80359151 |
| 116 | Yinchuan Shi | 7452.973741 | 106.340645 | 38.18184849 |
| 117 | Wuzhong Shi | 15866.27363 | 106.628695 | 37.4243355 |
| 118 | Qinzhou Shi | 10620.16925 | 109.02227 | 22.141575 |
| 119 | Zigong Shi | 4390.953531 | 104.658385 | 29.28437601 |
| 120 | Luzhou Shi | 12257.01679 | 106.3461212 | 28.49677881 |
| 121 | Neijiang Shi | 5405.425007 | 104.84807 | 29.61510249 |
| 122 | Nanchong Shi | 12578.2312 | 106.205375 | 31.15598598 |
| 123 | Guang'an Shi | 6369.499328 | 106.624485 | 30.43173498 |
| 124 | Heyuan Shi | 15654.19327 | 114.91237 | 23.975832 |
| 125 | Qingyuan Shi | 18966.94787 | 112.916635 | 24.33259401 |
| 126 | Zhaoqing Shi | 14924.29176 | 112.1145667 | 23.58688851 |
| 127 | Jiangmen Shi | 9383.618597 | 112.629275 | 22.27660101 |
| 128 | Shaoguan Shi | 18447.63718 | 113.79531 | 24.7044195 |
| 129 | Zhuhai Shi | 1522.440561 | 113.26147 | 22.15254201 |
| 130 | Xiangxi Tujiazu Miaozu Zizhizhou | 15500.76326 | 109.78151 | 28.68686001 |
| 131 | Zhangjiajie Shi | 9578.293881 | 110.510845 | 29.33470251 |
| 132 | Tongchuan Shi | 3931.589975 | 109.03542 | 35.1974205 |
| 133 | Guiyang Shi | 8058.215538 | 106.698875 | 26.773953 |
| 134 | Qiannan Buyizu Miaozu Zizhizhou | 26080.93468 | 107.25758 | 26.28452001 |
| 135 | Wuwei Shi | 32882.66779 | 103.01047 | 37.98502749 |
| 136 | Baiyin Shi | 20020.66354 | 104.536365 | 36.52372899 |
| 137 | Zhongwei Shi | 13192.58108 | 105.23042 | 36.94445112 |
| 138 | Guyuan Shi | 11340.42503 | 106.134705 | 35.93682066 |
| 139 | Changsha Shi | 11807.90411 | 113.070635 | 28.25664552 |
| 140 | Xiangtan Shi | 5053.119711 | 112.541125 | 27.71305449 |
| 141 | Loudi Shi | 8162.518064 | 111.642485 | 27.72306951 |
| 142 | Shizuishan Shi | 4104.022077 | 106.468235 | 39.00241848 |
| 143 | Huaihua Shi | 27586.1888 | 109.94658 | 27.444156 |
| 144 | Shaoyang Shi | 20860.07834 | 110.95405 | 26.81400201 |
| 145 | Changde Shi | 18294.46414 | 111.396755 | 29.2669665 |
| 146 | Yiyang Shi | 12192.46012 | 112.4337939 | 28.7533905 |
| 147 | Yueyang Shi | 14852.54993 | 113.23016 | 29.13753 |
| 148 | Binzhou Shi | 19421.33047 | 113.223835 | 25.867389 |
| 149 | Hengyang Shi | 15396.77439 | 112.408355 | 26.79209202 |
| 150 | Maoming Shi | 11388.51628 | 111.002605 | 22.0580685 |
| 151 | Sheng Zhixia Xingzhengdanwei | 29928.47406 | 109.82751 | 19.26870849 |
| 152 | Yulin Shi | 43136.52955 | 109.249135 | 38.199186 |
| 153 | Baoji Shi | 18136.43698 | 107.178195 | 34.339977 |
| 154 | Tianshui Shi | 14253.60771 | 105.64668 | 34.63386549 |
| 155 | Golog Zangzu Zizhizhou | 74484.04288 | 99.35481151 | 34.00672899 |
| 156 | Aba Zangzu Qiangzu Zizhizhou | 82820.87862 | 102.47673 | 32.45650299 |
| 157 | Garzee Zangzu Zizhizhou | 150134.5936 | 99.91979451 | 31.12478268 |
| 158 | Liangshan Yizu Zizhizhou | 60072.14597 | 101.96117 | 27.67700451 |
| 159 | Ya'an Shi | 15070.43511 | 102.66508 | 29.89311501 |
| 160 | Meishan Shi | 7224.710373 | 104.0949294 | 29.8788135 |
| 161 | Lupanshui Shi | 9948.377978 | 105.3932576 | 26.12637201 |
| 162 | Bijie Diqu | 26879.95687 | 105.158445 | 27.07010352 |
| 163 | Qiandongnan Miaozu Dongzu Zizhizhou | 30357.03389 | 108.429245 | 26.42575551 |
| 164 | Zunyi Shi | 30780.53298 | 106.912045 | 28.18140999 |
| 165 | Tongren Diqu | 17997.21377 | 108.6068 | 28.11157701 |
| 166 | Ziyang Shi | 7945.312754 | 104.96471 | 30.15918798 |
| 167 | Chengdu Shi | 12051.80757 | 103.95719 | 30.77060001 |
| 168 | Deyang Shi | 5984.671668 | 104.477625 | 31.11356451 |
| 169 | Xi'an Shi | 10210.50802 | 108.73968 | 34.21932999 |
| 170 | Foshan Shi | 3859.009401 | 112.88377 | 23.11270701 |
| 171 | Dongguan Shi | 2406.226286 | 113.8875731 | 22.8989475 |
| 172 | Shenzhen Shi | 1868.22807 | 114.195995 | 22.65221901 |
| 173 | Jieyang Shi | 5230.195418 | 116.11291 | 23.33154 |
| 174 | Shantou Shi | 2113.338218 | 116.520495 | 23.2800285 |
| 175 | Hainan Zangzu Zizhizhou | 42866.42472 | 100.3639035 | 35.89094349 |
| 176 | Bose Shi | 36318.08176 | 106.1677884 | 23.98296249 |
| 177 | Laibin Shi | 13385.23907 | 109.43045 | 23.86995201 |
| 178 | Liuzhou Shi | 18597.29474 | 109.377045 | 24.98067552 |
| 179 | Chongzuo Shi | 17495.05945 | 107.332975 | 22.47396549 |
| 180 | Nanning Shi | 22125.76846 | 108.47158 | 23.11916151 |
| 181 | Yulin Shi | 12876.12883 | 110.220665 | 22.384431 |
| 182 | Zhanjiang Shi | 12229.4604 | 110.315295 | 21.07601223 |
| 183 | Beihai Shi | 3360.392659 | 109.308745 | 21.658944 |
| 184 | Hohhot Shi | 17115.03457 | 111.40442 | 40.48552899 |
| 185 | Ulanqab Shi | 54607.29389 | 112.57587 | 41.77277901 |
| 186 | Wuhai Shi | 2925.573483 | 106.9159815 | 39.53617116 |
| 187 | Ordos Shi | 85522.53905 | 108.971225 | 39.24372099 |
| 188 | Haixi Mongolzu Zangzu Zizhizhou | 309928.7933 | 94.95728652 | 37.1093655 |
| 189 | Alxa Meng | 239314.4914 | 102.0146685 | 40.100445 |
| 190 | Jiuquan Shi | 167001.1859 | 96.29209117 | 40.48354569 |
| 191 | Haibei Zangzu Zizhizhou | 34364.68616 | 100.340648 | 37.91209398 |
| 192 | Zhangye Shi | 38446.45913 | 99.798421 | 38.69596701 |
| 193 | Huangnan Zangzu Zizhizhou | 17962.25913 | 101.565835 | 35.12363448 |
| 194 | Gannan Zangzu Zizhizhou | 36804.81021 | 102.760625 | 34.337196 |
| 195 | Dingxi Shi | 19703.10519 | 104.556615 | 35.08875999 |
| 196 | Lanzhou Shi | 13109.99318 | 103.648365 | 36.30045498 |
| 197 | Yongzhou Shi | 22387.55143 | 111.697495 | 25.75217802 |
| 198 | Hezhou Shi | 11688.05722 | 111.309975 | 24.39902751 |
| 199 | Fangchenggang Shi | 5925.021112 | 108.0159549 | 21.93407898 |
| 200 | Guangzhou Shi | 7198.618998 | 113.50522 | 23.336646 |
| 201 | Huizhou Shi | 11327.70386 | 114.614125 | 23.25423552 |
| 202 | Hulun Buir Shi | 234367.3479 | 120.80166 | 50.21188899 |
| 203 | Yushu Zangzu Zizhizhou | 197520.9538 | 93.58497251 | 33.91083399 |
| 204 | Wenzhou Shi | 11350.81118 | 120.43646 | 27.88911651 |
| 205 | Zhoushan Shi | 1224.749798 | 122.135875 | 30.06510048 |
| 206 | Fuzhou Shi | 11478.66327 | 119.167375 | 25.99103751 |
| 207 | Longyan Shi | 19126.56685 | 116.79186 | 25.2091875 |
| 208 | Ningde Shi | 13066.5248 | 119.491695 | 26.98505952 |
| 209 | Pingxiang Shi | 3800.796168 | 113.91814 | 27.48800952 |
| 210 | Xinyu Shi | 3202.869645 | 114.919475 | 27.8253375 |
| 211 | Yingtan Shi | 3528.201747 | 117.07367 | 28.24268802 |
| 212 | Ganzhou Shi | 39497.71645 | 115.27155 | 25.81751901 |
| 213 | Ji'an Shi | 25350.72148 | 114.87936 | 26.9723685 |
| 214 | Fuzhou Shi | 18884.22089 | 116.44215 | 27.50133999 |
| 215 | Qingdao Shi | 10830.68236 | 120.240825 | 36.36569199 |
| 216 | Yantai Shi | 13517.14956 | 120.738775 | 37.20543099 |
| 217 | Weifang Shi | 15721.7322 | 119.0922 | 36.5161095 |
| 218 | Weihai Shi | 5436.696055 | 121.937045 | 37.148472 |
| 219 | Rizhao Shi | 5256.261517 | 119.121415 | 35.56212051 |
| 220 | Linyi Shi | 17164.82734 | 118.302405 | 35.29359048 |
| 221 | Liaocheng Shi | 8593.236364 | 115.91051 | 36.40397649 |
| 222 | Luoyang Shi | 15122.06943 | 112.05606 | 34.32198948 |
| 223 | Sanmenxia Shi | 9925.206427 | 110.8217279 | 34.31634549 |
| 224 | Nanyang Shi | 26482.72548 | 112.391 | 33.03907548 |
| 225 | Xinyang Shi | 18931.28325 | 114.82415 | 32.02469199 |
| 226 | Zhumadian Shi | 15109.17434 | 114.139665 | 32.9071995 |
| 227 | Yuxi Shi | 15026.92083 | 102.21152 | 24.13382202 |
| 228 | Zhaotong Shi | 22543.30727 | 104.0884 | 27.60656649 |
| 229 | Honghe Hanizu Yizu Zizhizhou | 32151.60689 | 103.036955 | 23.60177799 |
| 230 | Wenshan Zhuangzu Miaozu Zizhizhou | 31535.30119 | 104.884735 | 23.56995552 |
| 231 | Xishuangbanna Daizu Zizhizhou | 19240.64719 | 100.8934745 | 21.86804451 |
| 232 | Dali Baizu Zizhizhou | 28444.51642 | 99.95574052 | 25.68441852 |
| 233 | Baoshan Shi | 19109.98087 | 99.070761 | 24.99022602 |
| 234 | Dehong Daizu Jingpozu Zizhizhou | 11186.05616 | 98.12887949 | 24.58819101 |
| 235 | Lijiang Shi | 20598.77699 | 100.4491085 | 26.957391 |
| 236 | Nujiang Lisuzu Zizhizhou | 14632.03446 | 98.89158651 | 26.972124 |
| 237 | Deqen Zangzu Zizhizhou | 23170.21325 | 99.45474851 | 28.06366101 |
| 238 | Lincang Diqu | 23713.33317 | 99.60873651 | 24.060693 |
| 239 | Lhasa Shi | 29617.77463 | 91.19177651 | 30.1498845 |
| 240 | Turpan Diqu | 69106.22207 | 89.59227 | 42.41210949 |
| 241 | Bortala Mongol Zizhizhou | 25007.45621 | 81.86837052 | 44.69837199 |
| 242 | Hotan Diqu | 249072.1027 | 81.15891299 | 37.00198749 |
| 243 | Altay Diqu | 117189.2726 | 88.300594 | 47.087406 |
| 244 | Fuyang Shi | 10096.11837 | 115.7502 | 32.9958765 |
| 245 | Haozhou Shi | 8576.114569 | 116.182315 | 33.47033898 |
| 246 | Nanping Shi | 26345.97986 | 118.135795 | 27.28455 |
| 247 | Zhangzhou Shi | 12534.07718 | 117.5174 | 24.39043002 |
| 248 | Chizhou Shi | 8477.167381 | 117.383925 | 30.20674701 |
| 249 | Wuhu Shi | 3355.888565 | 118.34217 | 31.08281547 |
| 250 | Nanchang Shi | 7408.992209 | 115.996135 | 28.67223414 |
| 251 | Jiujiang Shi | 18851.22864 | 115.41759 | 29.38666452 |
| 252 | Yichun Shi | 18692.15153 | 115.022945 | 28.328049 |
| 253 | Shangrao Shi | 22820.84795 | 117.35698 | 28.75413051 |
| 254 | Jingdezhen Shi | 5269.533747 | 117.329795 | 29.32936704 |
| 255 | Xuancheng Shi | 12233.70666 | 118.804675 | 30.63286098 |
| 256 | Huangshan Shi | 9799.109197 | 118.04795 | 29.96300556 |
| 257 | Huzhou Shi | 5822.552874 | 119.860275 | 30.78214548 |
| 258 | Lishui Shi | 17231.49844 | 119.565155 | 28.18509 |
| 259 | Huaibei Shi | 2735.257781 | 116.69387 | 33.6963252 |
| 260 | Suzhou Shi | 9943.812801 | 117.167165 | 33.96591399 |
| 261 | Hefei Shi | 7030.430009 | 117.28325 | 32.08517181 |
| 262 | Lu'an Shi | 18406.84571 | 116.29959 | 31.83443148 |
| 263 | Bengbu Shi | 5961.51075 | 117.40582 | 33.11140062 |
| 264 | Chuzhou Shi | 13481.44026 | 118.18904 | 32.53292049 |
| 265 | Jinhua Shi | 10935.20465 | 120.005185 | 29.10442449 |
| 266 | Quzhou Shi | 8856.876846 | 118.684265 | 28.869624 |
| 267 | Sanming Shi | 23056.1017 | 117.51202 | 26.303169 |
| 268 | Ma'anshan Shi | 1733.604028 | 118.6253818 | 31.54408548 |
| 269 | Jiaxing Shi | 4015.694783 | 120.784515 | 30.6797565 |
| 270 | Shaoxing Shi | 8015.248544 | 120.557975 | 29.7127539 |
| 271 | Hangzhou Shi | 16827.75666 | 119.5111667 | 29.87607099 |
| 272 | Taizhou Shi | 9350.801909 | 121.00192 | 28.7374755 |
| 273 | Ningbo Shi | 8567.151032 | 121.526655 | 29.7090675 |
| 274 | Anyang Shi | 7280.433359 | 114.7906473 | 35.7919635 |
| 275 | Hebi Shi | 2141.563793 | 114.360615 | 35.75178609 |
| 276 | Zhoukou Shi | 12020.21233 | 114.86505 | 33.70056699 |
| 277 | Shangqiu Shi | 10732.30554 | 115.7357 | 34.28940798 |
| 278 | Xuchang Shi | 5005.288759 | 113.68759 | 34.04979498 |
| 279 | Jiyuan Shi | 1888.378177 | 112.393625 | 35.09120151 |
| 280 | Luohe Shi | 2642.411145 | 113.874155 | 33.700119 |
| 281 | Pingdingshan Shi | 7963.781111 | 112.959745 | 33.74392899 |
| 282 | Puyang Shi | 4233.821308 | 115.48773 | 35.77579299 |
| 283 | Jiaozuo Shi | 4138.537105 | 113.09015 | 35.15780451 |
| 284 | Zhengzhou Shi | 7459.111542 | 113.458645 | 34.62163398 |
| 285 | Kaifeng Shi | 6232.016712 | 114.56757 | 34.60871298 |
| 286 | Xinxiang Shi | 8328.30147 | 114.20333 | 35.35729749 |
| 287 | Laiwu Shi | 2186.803094 | 117.65393 | 36.27688398 |
| 288 | Dezhou Shi | 10300.88776 | 116.677245 | 37.20577248 |
| 289 | Dongying Shi | 6822.889621 | 118.66206 | 37.54125399 |
| 290 | Zibo Shi | 6025.714015 | 118.02929 | 36.620943 |
| 291 | Binzhou Shi | 8607.080424 | 117.822365 | 37.48372998 |
| 292 | Zaozhuang Shi | 4542.469128 | 117.32166 | 34.89577098 |
| 293 | Jining Shi | 11145.74495 | 116.72497 | 35.21690949 |
| 294 | Heze Shi | 12159.09149 | 115.602175 | 35.20914849 |
| 295 | Jinan Shi | 8040.285404 | 116.96996 | 36.781605 |
| 296 | Tai'an Shi | 7681.163826 | 117.016975 | 36.05735601 |
| 297 | Puer Shi | 44256.58088 | 100.751733 | 23.42345502 |
| 298 | Tacheng Diqu | 95152.4658 | 85.45064978 | 45.28683999 |
| 299 | Aksu Diqu | 127108.6839 | 81.06001701 | 41.06807349 |
| 300 | Kizilsu Kirgiz Zizhizhou | 72412.86604 | 76.162029 | 39.60809298 |
| 301 | Shannan Diqu | 79328.36105 | 92.212761 | 28.3402965 |
| 302 | Nyingchi Diqu | 115252.8431 | 95.45892001 | 29.11054101 |
| 303 | Qamdo Diqu | 109134.6623 | 96.35491149 | 30.51315237 |
| 304 | Xigaze Diqu | 180258.9453 | 86.24591401 | 29.51644002 |
| 305 | Kunming Shi | 21071.65098 | 102.921075 | 25.46148849 |
| 306 | Huainan Shi | 2647.743084 | 116.78282 | 32.69755371 |
| 307 | Shiyan Shi | 23643.70819 | 110.50688 | 32.39670099 |
| 308 | Xiangfan Shi | 19738.872 | 111.93855 | 31.93635348 |
| 309 | Jingmen Shi | 12360.1368 | 112.668955 | 31.00673748 |
| 310 | Huanggang Shi | 17440.08479 | 115.265045 | 30.6659985 |
| 311 | Enshi Tujiazu Miaozu Zizhizhou | 24201.0771 | 109.50309 | 30.25671099 |
| 312 | Xiaogan Shi | 8886.994356 | 113.945915 | 31.23349548 |
| 313 | Suizhou Shi | 9605.813306 | 113.42321 | 31.88602449 |
| 314 | Sheng Zhixia Xingzhengdanwei | 10429.41531 | 113.164725 | 30.48872499 |
| 315 | Xianning Shi | 9744.299496 | 114.252095 | 29.66735049 |
| 316 | Jingzhou Shi | 14080.95421 | 112.66644 | 30.05135901 |
| 317 | Wuhan Shi | 8540.117535 | 114.386565 | 30.67497051 |
| 318 | Huangshi Shi | 4570.299936 | 115.006055 | 29.92392945 |
| 319 | Ezhou Shi | 1593.025773 | 114.7922 | 30.31916448 |
| 320 | Yichang Shi | 21317.38659 | 111.17539 | 30.76347549 |
| 321 | Xiamen Shi | 1556.380191 | 118.117345 | 24.67400988 |
| 322 | Nagqu Diqu | 376171.701 | 89.44913851 | 33.213762 |
| 323 | Ngari Diqu | 314345.1755 | 82.37661884 | 32.70066849 |
| 324 | Kashi(Kaxgar) Diqu | 109810.9544 | 77.16882752 | 37.86136449 |
| 325 | Zizhiqu Zhixia Xingzhengdanwei | 7492.360181 | 81.32339232 | 40.64038038 |
| 326 | Qujing Shi | 28915.71777 | 103.93697 | 25.70294301 |
| 327 | Chuxiong Yizu Zizhizhou | 28410.09288 | 101.6029 | 25.364868 |
| 328 | Bayingolin Mongol Zizhizhou | 468923.4775 | 88.225014 | 39.59387853 |
| 329 | Hami(Kumul) Diqu | 137083.0504 | 93.7514155 | 42.9762495 |
| 330 | Chongqing Shi | 82538.0908 | 107.738905 | 30.18808548 |
| 331 | Panzhihua Shi | 7420.037605 | 101.691185 | 26.71933905 |
| 332 | Xianyang Shi | 10245.12573 | 108.408525 | 34.87574001 |
| 333 | Urumqi Shi | 11625.72895 | 87.880943 | 43.69864185 |
| 334 | Changji Huizu Zizhizhou | 76287.98111 | 88.42097101 | 44.294571 |
| 335 | Quanzhou Shi | 10940.43023 | 118.29883 | 25.19196222 |
| 336 | Putian Shi | 3756.639655 | 118.909635 | 25.43385702 |
| 337 | Anqing Shi | 15423.72395 | 116.74529 | 30.52641948 |
| 338 | Tongling Shi | 1036.909253 | 117.92746 | 30.94092648 |
| 339 | Karamay Shi | 7776.282396 | 84.99553269 | 45.43084149 |
| 340 | Ili Kazak Zizhizhou | 56231.12114 | 82.56569702 | 43.55294799 |
