# Supplementary figures and images for: Spatial and temporal clustering analysis of tuberculosis in the mainland of China at the prefecture level, 2005–2015
Source: Infect Dis Poverty. 2018 Oct 20;7:106. doi: 10.1186/s40249-018-0490-8 (PMC6195697; doi:10.1186/s40249-018-0490-8)

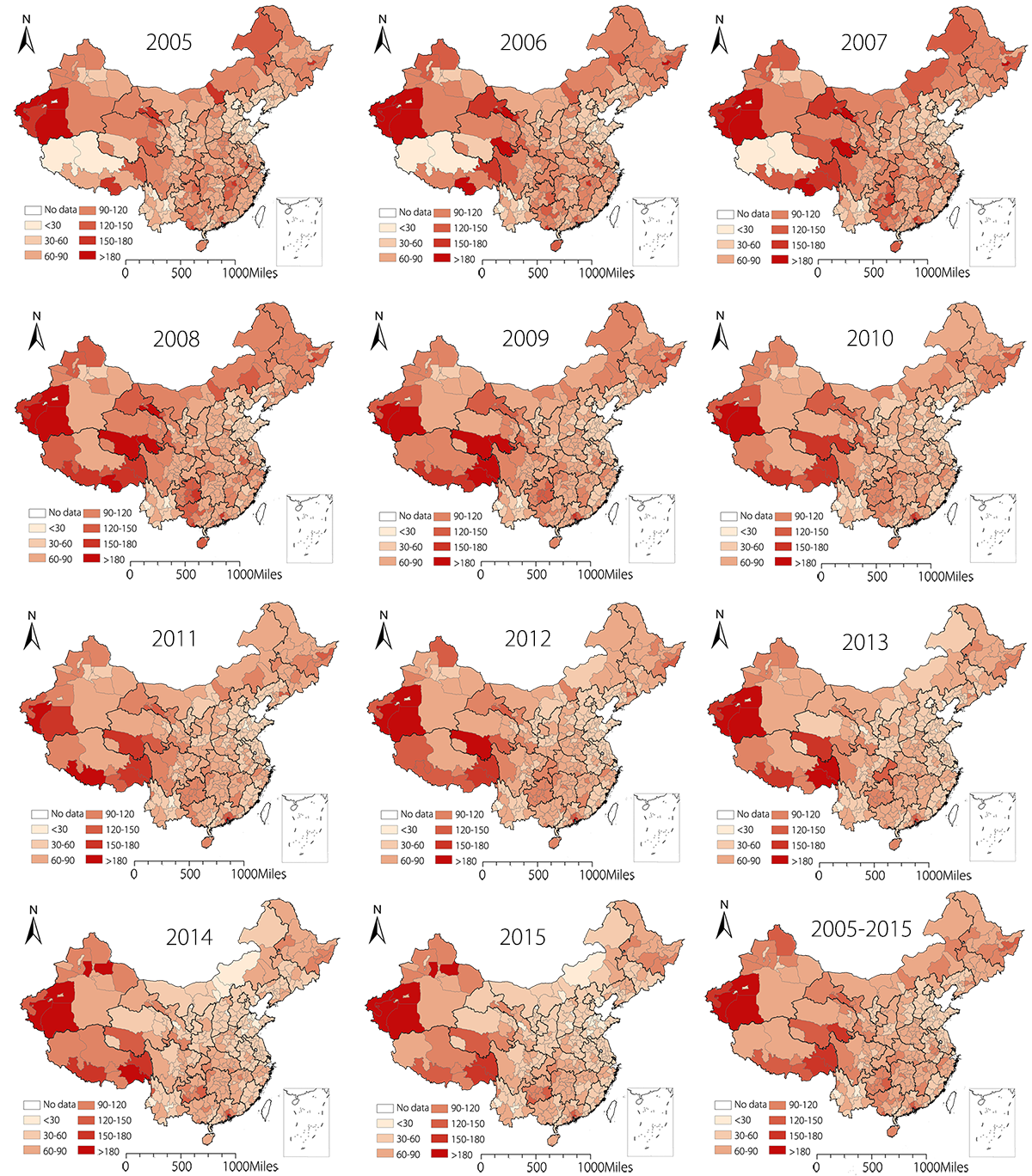

Supplement: Supplementary file 3 — The reported tuberculosis incidence of 340 prefectures in the mainland of China from 2005 to 2015 (TIF 15157 kb) [file 40249_2018_490_MOESM3_ESM.tif]
