# Supplementary material for: Spatial and temporal clustering analysis of tuberculosis in the mainland of China at the prefecture level, 2005–2015
Source: Infect Dis Poverty. 2018 Oct 20;7:106. doi: 10.1186/s40249-018-0490-8 (PMC6195697; doi:10.1186/s40249-018-0490-8)
Supplement: Supplementary file 4 — Spatio-temporal clustering of sputum smear-positive tuberculosis in the mainland of China from 2005 to 2015. (DOCX 16 kb) [file 40249_2018_490_MOESM4_ESM.docx]

Additional Table S2. Spatio-temporal clustering of sputum smear-positive tuberculosis in the mainland of China from 2005 to 2015

| Cluster type | Cluster time frame | Coordinates/ Radius | N | Observed cases | Expected cases | *RR* | *LLR* | *P*-value |
| --- | --- | --- | --- | --- | --- | --- | --- | --- |
| Most likely cluster | 1 March 2005‒31 May 2008 | (41.068073 N, 81.060017 E) / 628.47 km | 10 | 46859 | 15016.12 | 3.14 | 21593.60 | <0.001 |
| Secondary cluster1 | 1 March 2005‒31 May 2008 | (30.051359 N, 112.666440 E) / 357.02 km | 31 | 230720 | 147128.06 | 1.60 | 20993.28 | <0.001 |
| Secondary cluster2 | 1 March 2005‒31 May 2008 | (51.862772 N, 124.098025 E) / 1064.00 km | 26 | 124682 | 79818.46 | 1.58 | 10967.90 | <0.001 |
| Secondary cluster3 | 1 March 2007‒31 May 2010 | (30.770600 N, 103.957190 E) / 394.51 km | 24 | 193351 | 137037.43 | 1.43 | 10602.36 | <0.001 |
| Secondary cluster4 | 1 March 2005‒31 May 2008 | (22.152542 N, 113.261470 E) / 494.37 km | 35 | 199865 | 142812.44 | 1.42 | 10488.16 | <0.001 |
| Secondary cluster5 | 1 March 2005‒31 May 2008 | (32.532920 N, 118.189040 E) / 270.93 km | 29 | 187282 | 140504.78 | 1.35 | 7287.20 | <0.001 |
| Secondary cluster6 | 1 April 2005‒30 June 2005 | (35.751786 N, 114.360615 E) / 261.04 km | 25 | 18387 | 10555.62 | 1.74 | 2379.78 | <0.001 |
| Secondary cluster7 | 1 February 2006‒30 April 2009 | (41.400669 N, 117.569510 E) / 0 km | 1 | 5850 | 3672.43 | 1.59 | 546.64 | <0.001 |
| Secondary cluster8 | 1 February 2006‒30 April 2009 | (39.745525 N, 118.408410 E) / 74.76 km | 2 | 12835 | 10502.25 | 1.22 | 242.36 | <0.001 |

Most likely cluster: *P* value<0.001; Secondary cluster: *P* value<0.001;

RR: relative risk; N: number of prefectures in the cluster.
