# Supplementary material for: Spatial and temporal clustering analysis of tuberculosis in the mainland of China at the prefecture level, 2005–2015
Source: Infect Dis Poverty. 2018 Oct 20;7:106. doi: 10.1186/s40249-018-0490-8 (PMC6195697; doi:10.1186/s40249-018-0490-8)
Supplement: Supplementary file 5 — Spatio-temporal clustering of sputum smear-negative tuberculosis in the mainland of China from 2005 to 2015. (DOCX 19 kb) [file 40249_2018_490_MOESM5_ESM.docx]

Additional Table S3. Spatio-temporal clustering of sputum smear-negative tuberculosis in the mainland of China from 2005 to 2015

| Cluster type | Cluster time frame | Coordinates/ Radius | N | Observed cases | Expected cases | *RR* | *LLR* | *P*-value |
| --- | --- | --- | --- | --- | --- | --- | --- | --- |
| Most likely cluster | 1 November 2012‒31 December 2015 | (39.608093 N, 76.162029 E) / 523.07 km | 5 | 55843 | 11406.05 | 4.94 | 44451.88 | <0.001 |
| Secondary cluster1 | 1 February 2012‒30 April 2015 | (26.425756 N, 108.429245 E) / 479.85 km | 38 | 351976 | 232381.86 | 1.55 | 27966.59 | <0.001 |
| Secondary cluster2 | 1 June 2009‒31 August 2012 | (22.898947 N, 113.887573 E) / 105.39 km | 7 | 66166 | 26145.90 | 2.55 | 21565.88 | <0.001 |
| Secondary cluster3 | 1 November 2012‒31 December 2015 | (47.952791 N, 129.199285 E) / 374.92 km | 12 | 74738 | 42777.54 | 1.76 | 9839.16 | <0.001 |
| Secondary cluster4 | 1 March 2006‒31 May 2009 | (29.104424 N, 120.005185 E) / 186.96 km | 9 | 74888 | 49924.36 | 1.51 | 5461.92 | <0.001 |
| Secondary cluster5 | 1 March 2006‒31 May 2009 | (39.813770 N, 98.164089 E) / 364.59 km | 6 | 11319 | 3972.55 | 2.85 | 4510.49 | <0.001 |
| Secondary cluster6 | 1 March 2005‒31 August 2007 | (28.328049 N, 115.022945 E) / 177.35 km | 9 | 40995 | 28564.79 | 1.44 | 2394.98 | <0.001 |
| Secondary cluster7 | 1 February 2012‒30 April 2015 | (33.039075 N, 112.391000 E) / 307.00 km | 25 | 172611 | 147534.41 | 1.18 | 2081.04 | <0.001 |
| Secondary cluster8 | 1 August 2010‒31 October 2013 | (41.195207 N, 124.682045 E) / 0 km | 1 | 4868 | 1819.55 | 2.68 | 1743.00 | <0.001 |
| Secondary cluster9 | 1 March 2006‒31 May 2009 | (44.177277 N, 115.525760 E) / 275.36 km | 2 | 11525 | 6373.35 | 1.81 | 1678.20 | <0.001 |
| Secondary cluster10 | 1 May 2006‒30 November 2008 | (32.085172 N, 117.283250 E) / 0 km | 1 | 8885 | 4746.58 | 1.87 | 1433.56 | <0.001 |
| Secondary cluster11 | 1 March 2005‒31 May 2008 | (41.772779 N, 112.575870 E) / 0 km | 1 | 5017 | 2536.83 | 1.98 | 941.59 | <0.001 |
| Secondary cluster12 | 1 March 2007‒31 July 2009 | (39.536171 N, 106.915981 E) / 395.02 km | 13 | 25746 | 20225.50 | 1.27 | 695.81 | <0.001 |
| Secondary cluster13 | 1 February 2012‒30 April 2015 | (35.775793 N, 115.487730 E) / 62.87 km | 2 | 15009 | 11334.58 | 1.33 | 541.26 | <0.001 |
| Secondary cluster14 | 1 December 2006‒30 September 2009 | (41.156280 N, 123.131015 E) / 0 km | 1 | 3388 | 1887.97 | 1.80 | 481.27 | <0.001 |
| Secondary cluster15 | 1 June 2005‒31 August 2008 | (39.891544 N, 113.555935 E) / 0 km | 1 | 5436 | 3638.55 | 1.49 | 385.18 | <0.001 |
| Secondary cluster16 | 1 May 2013‒31 December 2015 | (40.597206 N, 120.123700 E) / 0 km | 1 | 3957 | 2720.30 | 1.45 | 246.30 | <0.001 |
| Secondary cluster17 | 1 November 2011‒31 December 2011 | (33.528506 N, 120.184190 E) / 185.08 km | 8 | 3588 | 2682.86 | 1.34 | 138.01 | <0.001 |
| Secondary cluster18 | 1 March 2005‒31 August 2006 | (33.696325 N, 116.693870 E) / 0 km | 1 | 1754 | 1152.69 | 1.52 | 135.06 | <0.001 |
| Secondary cluster19 | 1 March 2015‒31 July 2015 | (41.400669 N, 117.569510 E) / 0 km | 1 | 932 | 581.16 | 1.60 | 89.36 | <0.001 |
| Secondary cluster20 | 1 December 2009‒31 December 2009 | (38.896553 N, 112.450270 E) / 0 km | 1 | 244 | 95.32 | 2.56 | 80.67 | <0.001 |
| Secondary cluster21 | 1 March 2010‒30 June 2010 | (34.331669 N, 117.512545 E) / 0 km | 1 | 1577 | 1172.37 | 1.35 | 62.96 | <0.001 |
| Secondary cluster22 | 1 November 2015‒31 December 2015 | (37.709760 N, 115.869995 E) / 0 km | 1 | 451 | 274.74 | 1.64 | 47.28 | <0.001 |
| Secondary cluster23 | 1 April 2014‒31 July 2014 | (38.098423 N, 114.494145 E) / 0 km | 1 | 1570 | 1238.98 | 1.27 | 40.74 | <0.001 |

Most likely cluster: *P* value<0.001; Secondary cluster: *P* value<0.001;

RR: relative risk; N: number of prefectures in the cluster.
